# Aberrant myonuclear domains and impaired myofiber contractility despite marked hypertrophy in *MYMK*-related, Carey-Fineman-Ziter Syndrome

**DOI:** 10.1186/s40478-024-01783-2

**Published:** 2024-05-24

**Authors:** Hannah F. Dugdale, Yotam Levy, Heinz Jungbluth, Anders Oldfors, Julien Ochala

**Affiliations:** 1 School of Sport, Exercise and Health Sciences, Loughborough University, Loughborough, UK; 2 Centre for Human and Applied Physiological Sciences, School of Basic and Medical Biosciences, Faculty of Life Sciences and Medicine, King’s College London, London, UK; 3 Randall Centre for Cell and Molecular Biophysics, Muscle Signalling Section, Faculty of Life Sciences and Medicine (FoLSM), King’s College London, London, UK; 4 Department of Paediatric Neurology, Neuromuscular Service, Evelina Children’s Hospital, Guy’s and St Thomas’ Hospital NHS Foundation Trust, London, UK; 5 Department of Laboratory Medicine, University of Gothenburg, Gothenburg, Sweden; 6 Department of Biomedical Sciences, Faculty of Health and Medical Sciences, University of Copenhagen, Copenhagen, Denmark

**Keywords:** *MYMK* gene, Myomaker, Skeletal muscle, Myonuclear domain, Single myofiber, Carey-Fineman-Ziter Syndrome

## Abstract

Carey Fineman Ziter Syndrome (CFZS) is a rare autosomal recessive disease caused by mutations in the *MYMK* locus which encodes the protein, myomaker. Myomaker is essential for fusion and concurrent myonuclei donation of muscle progenitors during growth and development. Strikingly, in humans, *MYMK* mutations appear to prompt myofiber hypertrophy but paradoxically, induce generalised muscle weakness. As the underlying cellular mechanisms remain unexplored, the present study aimed to gain insights by combining myofiber deep-phenotyping and proteomic profiling. Hence, we isolated individual muscle fibers from CFZS patients and performed mechanical, 3D morphological and proteomic analyses. Myofibers from CFZS patients were ~ 4x larger than controls and possessed ~ 2x more myonuclei than those from healthy subjects, leading to disproportionally larger myonuclear domain volumes. These greater myonuclear domain sizes were accompanied by smaller intrinsic cellular force generating-capacities in myofibers from CFZS patients than in control muscle cells. Our complementary proteomic analyses indicated remodelling in 233 proteins particularly those associated with cellular respiration. Overall, our findings suggest that myomaker is somewhat functional in CFZS patients, but the associated nuclear accretion may ultimately lead to non-functional hypertrophy and altered energy-related mechanisms in CFZS patients. All of these are likely contributors of the muscle weakness experienced by CFZS patients.

**Supplementary Information:**

The online version contains supplementary material available at 10.1186/s40478-024-01783-2.

## Introduction

Carey-Fineman-Ziter Syndrome (CFZS) is a rare autosomal-recessive disorder often classified as a congenital myopathy. CFZS is characterised by facial dysmorphisms and motor developmental delays associated with distinct skeletal muscle symptoms [5, 6]. The condition shares close similarities with other dysmorphic syndromes with skeletal muscle involvement, in particular King-Denborough syndrome (KDS) and Noonan syndrome (NS), both of which have to be considered in the differential diagnosis. The prevalence of this *MYMK*-related disease is unclear due, in part, to underrecognition, particularly when dysmorphic features are subtle. Hence, to date, confirmation of a CFZS diagnosis has been documented in less than twenty patients [1, 9, 18]. A total of seven missense *MYMK* mutations have been identified, of which two have been classified as hypomorphic (c.271 C > A and c.461T > C), three as null (c.553T > C, c.298G > A and c.2T > A) and two are yet to be categorised (c.235T > C and c.399 + 5G > A) [1, 4, 9, 18]. More recently, a CFZS-like phenotype has also been documented due to mutation in another myoblast fusion gene, *MYMX* (c.136 C > T) [33]. Irrespective of mutation, CFZS patients all exhibit muscle weakness. Interestingly, while lower limb MRIs indicate that some fat infiltration occurs, overall muscle mass is similar to controls [9]. Surprisingly, histological analysis reveals striking myofiber hypertrophy leading to the inference that patients likely possess 75% fewer fibers than healthy individuals [18]. Despite this knowledge the exact pathophysiology underlying these muscle symptoms is not yet elucidated. Hence, the aim of the present study was to characterise the cellular mechanisms by which CFZS-associated muscle weakness occurs in the presence of significant hypoplasia and concomitant myofiber hypertrophy.

Muscle fiber hypertrophy has been described in other genetic muscular disorders including, but not limited to; Duchenne Muscular Dystrophy (DMD) and Spinal Muscular Atrophy (SMA). In DMD, while imaging studies have identified true hypertrophy in some individuals, it is typically classified as pseudo-hypertrophy due to the enlarged outer appearance, resultant from the presence of oedema and extensive fat and connective tissue infiltration [20]. In SMA, muscle fiber hypertrophy is sparse and organised in clusters, while most fibers are atrophied [19]. Despite these observations, decreases in contractile material and progressive gross atrophy inevitably leads to overall muscle weakness in DMD and SMA, however, as this is not the case in CFZS alternative causes for the induction of muscle weakness are likely.

The *MYMK* gene encodes a highly conserved muscle specific protein named myomaker. Myomaker is essential for fusion and concurrent myonuclei donation of muscle progenitors during growth and development [25, 27]. Intriguingly, while human *MYMK* mutations prompt myofiber hypertrophy in CFZS, *MYMK* knockout (KO) mutations in zebrafish or mice prevent myonuclear accretion [26, 39] and produce an abrogated hypertrophic response to physiological stimuli [14, 15]. This indicates the presence of patient specific cellular mechanisms that we aim to address in the present work.

Healthy skeletal muscle fibers contain several hundred peripherally located and evenly distributed myonuclei [13, 38]. Indeed, following fusion, each myonucleus controls transcriptional activity for a well-defined volume of cytoplasm, termed the myonuclear domain (MND) [8, 11, 16, 30]. As such, sufficient peripheral nuclear number and regular positioning are crucial for correct myonuclear functioning as well as proper myofibril growth and contractility [36]. Reducing or altering myonuclear accretion and extending MND volumes beyond their maximum functional capacities leads to pathological states and depressed muscle fiber force production [21]. We therefore initially hypothesized that CFZS-associated *MYMK* mutations produce dysfunctional myomaker proteins. The nuclear accretion may in turn be ineffective, resulting in hypertrophic myofibers with disproportionally large dysfunctional MND sizes and compromised intrinsic cellular force production, all of which ultimately contributes to overall muscle weakness reported in CFZS patients. To investigate these hypotheses, in the present study we isolated and membrane-permeabilised individual muscle fibers from *MYMK-*mutated human muscle tissue and performed a deep phenotyping consisting of a series of mechanical and morphological analyses, including an evaluation of the 3D organisation of myonuclei. In parallel, we ran a proteomics analysis to detect potential clusters of proteins preferentially dysregulated in myofibers from the patients.

## Materials and methods

### Human participants

Two siblings (one male, aged 6 years and one female, aged 18 years) carrying autosomal-recessive mutation in the myomaker gene (*MYMK/TMEM8C*) [c.235T > C; p.(Trp79Arg), and biopsies from the vastus lateralis in compliance with the declaration of Helsinki, as previously described by [18]. Seven vastus lateralis muscle biopsies from healthy donors were also included in the present study (Table 1). All specimens were snap-frozen in liquid propane chilled with liquid nitrogen and stored at – 80ºC until required for further analysis. All tissue was consented, stored, and used in accordance with the Human Tissue Act, UK, under local ethical approval (REC 13/NE/0373).

**Table 1:** Patient and control muscle biopsy samples used

Age (years)	Gender	Mutation	Disease
6	Male	*MYMK*/*TMEM8C*) [c.235T > C; p.(Trp79Arg), and c.271 C > A; p.(Pro91Thr)],	Congenital myopathy
18	Female	*MYMK/TMEM8C*) [c.235T > C; p.(Trp79Arg), and c.271 C > A; p.(Pro91Thr)],	Congenital myopathy
25	Female	-	-
37	Male	-	-
21	Female	-	-
24	Female	-	-
35	Female	-	-
42	Male	-	-
27	Male	-	-

### Muscle fiber permeabilization

Muscle samples were defrosted, placed in relaxing solution at 4 °C followed by skinning solution (relaxing solution containing glycerol) for 24 h at 4 °C, after which they were transferred to -20 °C for short term storage (3 to 4 weeks). For long-term storage the muscle bundles were treated with sucrose, a cryoprotectant, within 1–2 weeks [12] and snap frozen in liquid nitrogen-chilled propane, and stored at − 80 °C.

### Solutions

Relaxing and activating solutions contained 4 mM Mg-ATP mM free Mg^2+^, 20 mM imidazole, 7 mM EGTA, 14.5 mM creatine phosphate and KCl to adjust ionic strength to 180 mM and pH to 7.0. The concentrations of free Ca^2+^ was 10^− 9^ M for the relaxing buffer (pCa 9) and 10^− 4.5^ for the activating solution (pCa 4.5). Skinning solution contain relaxing solution supplemented with glycerol (50:50 v/v).

### Single fiber isolation

Single myofibers were manually dissected at room temperature (RT). For image analysis, each end of the dissected myofiber was clamped to half split copper meshes designed for electron microscopy (SPI G100 2010 C-XA, width 3 mm) which had been glued to coverslips (Menzel-Gläser, 22 × 50 mm, thickness 0.13–0.16 mm), with each array containing approximately nine myofibers. Approximately 16 fibers were tested for each participant, therefore a total of 114 fibers from seven participants and 32 fibers from two participants were analysed for CON and CFZS respectively.

### Single myofiber force production

Individual fibers were attached between connectors leading to a force transducer (model 400 A; Aurora Scientific) and a lever arm system (model 308B; Aurora Scientific). Sarcomere length was set to approximately 2.50 μm, while the temperature was set to 15 °C [23, 24, 29]. Absolute maximal isometric force generation was calculated as the difference between total tension in the activating solution (pCa 4.5) and the resting tension for the same myofiber in the relaxing solution (pCa 9.0). Specific force was defined as absolute force divided by CSA (estimated from the width and depth, assuming an elliptical circumference). Myofibers included in the analysis were able to sustain three consecutive maximal activations without any force depressions (> 10%) and had preserved sarcomere structures after the three maximal activations. Approximately 7–9 fibers were used per individual for the control group (*n* = 40) and > 10 individual fibers per CFZS patient (*n* = 25) sample groups,

### Fluorescence labelling

At room temperature, arrays were fixed in 4% PFA for 15 min (minutes), washed 3 times with PBS and further permeabilized in 0.1% Triton-X100/PBS for 10 min. Subsequently fibers were blocked using 10% goat serum (GS) for 1 h. Myofibers were incubated in primary antibodies Desmin [D33], ab8470 (Abcam) (1:150) overnight. The myofibers were then washed again in PBS before incubation with associated secondary antibody [FluorTM 488 Goat anti-Rabbit IgG H + L, A-11,008 (Thermo Fisher Scientific)] and/or DAPI.

### Fluorescence imaging and analysis

A Confocal microscope (Zeiss Axiovert 200, objectives ×20 and ×40) equipped with a CARV II confocal imager (BD Biosciences) was used to acquire images. To visualize muscle fibers in 3D, stacks of 200 images were acquired (1 μm Z increments) and analysed with a custom-made MATLAB (MathWorks) program [21]. To measure how ordered the nuclear distribution for a particular fiber experimental NN (nearest neighbour) distances were calculated and compared to simulated distributions: a theoretical optimal and a theoretical random distribution. We denoted experimental, optimal and random distributions by *M*_*E*_, *M*_*o*_ and *M*_*R*_ respectively. An order score g [3], was then calculated using the following equation: *g = (M*_*E*_*- M*_*R*_*) / (M*_*O*_*– M*_*R*_*).*

### LC-MS / MS identification and quantitative analysis of protein abundance

For preparation, each sample consisted of five individual muscle fibers which were dissected at a length of 3 mm. A total of five samples were produced for each of the two control participants (*n* = 10) and each of the two CFZS patients (*n* = 10). Samples were provided in a centrifuge tube containing 30 µl Tris-Triton lysis buffer (10 mM Tris, pH 7.4, 100 mM NaCl, 1 mM EDTA, 1 mM EGTA, 1% Triton X-100, 10% glycerol, 0.1% SDS, 0.5% deoxycholate, protease inhibitor cocktail III (1:100), phosphatase inhibitor cocktail mix (1:100) at an unknown protein concentration. Sample volume was reduced by half in a SpeedVac (ThermoFisherScientific) and subsequently mixed in a 1:1 ratio with Laemmli buffer (2x conc.), vortexed and boiled at 96 °C for 10 min. To stack the protein complement and remove chemical interference from the lysis buffer samples were centrifuged at 14,000 rpm for 3 min prior to loading in 10% BisTris gels (Gel 1 - #19072670-1957; Gel 2 - #19072670-1965; Gel 3 - #19072670-1966; Gel 4 - #19072670-1977). Gels were then stained overnight with Imperial protein stain (Thermo, #24,615). In-gel reduction, alkylation and digestion with trypsin was performed prior to subsequent isobaric mass tag labelling [10]. Each sample was treated individually with labels (TMT10plex) added at a 1:1 ratio.

For analysis by LC-MS/MS, TMT labelled peptide samples were resuspended in 60 µL of resuspension buffer (2% ACN in 0.05% FA) with 10 µl sample injected in triplicate (30 µl total volume). Chromatographic separation was performed using an Ultimate 3000 NanoLC system (ThermoFisherScientific, UK). Peptides were resolved by reversed phase chromatography on a 75 μm * 50 cm C18 column using a three-step gradient of water in 0.1% formic acid and 80% acetonitrile in 0.1% formic acid. The gradient was delivered to elute the peptides at a flow rate of 250 nl/ min over 250 min. The eluate was ionised by electrospray ionisation using an Orbitrap Fusion Lumos (ThermoFisherScientific, UK) operating under Xcalibur v4.1. The instrument was programmed to acquire using a “Synchronous Precursor Selection with MultinotchMS^3^” method (SPS). Synchronous Precursor Selection is a process of selecting multiple MS2 precursors using a single fill and single waveform in a CID or HCD cell, while MultinotchMS3 is to reduce co-isolated interference from MS2 in an ion-trap cell.

### Fiber type estimation

Previously defined by Murgia et al. [28] for each sample the abundance of Myosin heavy chains 1,2,4 and 7 (MYH1,2,4 and 7) was summed and the expression of each isoform was then calculated as a percentage. Pure fibers were expressed ≥ 80% of a particular MHY protein, for example those samples possessing ≥ 80% of MYH7 were classified as a pure slow fiber. Where the 80% expression threshold was not met the fiber was classified as a mixed fiber. Here the highest and second highest percentage contribution providing the classification i.e. MYH7 and MHY2 classified as a Mixed slow/2A fiber. Each sample group contained five individual fibers; therefore, the fiber type is estimated based on the relative myosin expression for each sample, not each fiber.

### Database searching

Raw mass spectrometry data from the triplicate injection were processed into peak list files using Proteome Discoverer (ThermoScientific; v2.2) (PD 2.2). The data was processed and searched using the Mascot search algorithm (v2.6.0; www.matrixscience.com) and the Sequest search algorithm [10] against the Uniprot Human Taxonomy database (49,806 entries; http://www.uniprot.org/uniprot/). Within the consensus processing module, the reporter ion intensity values (absolute area under the peak) for each peptide spectral match are grouped with peptides and calculated at the protein level identification as a grouped abundance. All grouped abundances at protein level are normalised using total peptide amount which has previously been corrected based on the highest peptide abundance present in one channel, thus all channels have the same total abundance.

### Bioinformatics and data visualisations

Following processing with Proteome Discoverer, the result file was exported into Perseus (v1.6.3; http://www.perseus-framework.org) for qualitative and quantitative data analysis (two sample, student t-test with permutation based false discovery rate [FDR]). Metascape [40] was utilised for gene ontological (GO) analysis, which was subsequently visualised using cytoscape [37]. Control and patient mean protein abundance was used to visualise composition of protein functions using an automatic system based on KEGG Pathways gene classifications, named proteomaps [22]. Further data visualisation utilised Biovinci (v3.0.9; https://vinci.bioturing.com) and Graphpad Prism (v9;.www.graphpad.com).

### Statistics

Statistical analyses were performed using GraphPad Prism 6.0 and included normality tests, t-tests/Mann-Whitneys, ANOVAs and subsequent post-hoc tests, while Pearson’s product moment correlation (to evaluate linear relationships) and comparison between regression coefficients were carried out on SPSS statistics 23 software (IBM). Principal component analysis (PCA) and Pearson correlations for fiber typing was carried out and visualised using Biovinci (v3.0.9; https://vinci.bioturing.com). Statistical significance was set to *p* < 0.05. Parametric data are presented as mean ± SD whereas non-parametric data sets are presented using median ± 95% confidence intervals.

## Results

### Marked cellular hypertrophy in CFZS patients is confirmed via 3D analysis of single myofibers

**Fig. 1:**
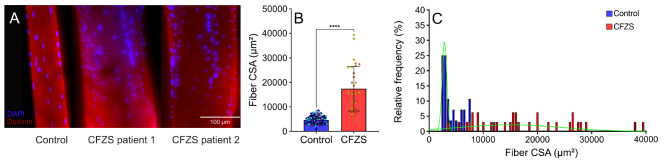
**Fiber size comparison between control and CFZS patients.** (**A**) Representative images of a single fiber from a control and from each of the CFZS patients (114 fibers from seven controls and 32 fibers from two CFZS patients) red is desmin and blue is dapi (**B**) Fiber CSA for controls and CFZS patients. (**C**) Relative frequency of Fiber CSA is presented as median ± interquartile range and significance was determined by Mann Whiney test, **** represents a p value of < 0.0001

Significant myofiber hypertrophy was previously documented in CFZS patients through histological 2D measurements [9, 18]. To confirm these findings in a more physiologically accurate manner, single muscle fibers were manually isolated from the biopsies of patients diagnosed with Carey-Fineman-Ziter-Syndrome (CFZS) and their control counterparts (CON). In the current experimentation we did not delineate fiber type as previous histological analysis of these patient samples (taken from the same muscle) suggested all fiber types underwent significant myofiber hypertrophy, thus providing us with an increased number of fibers available for downstream analysis [18]. These fibers were stained for nuclei (DAPI) and myofibrils (Desmin) from which, subsequent 3D confocal reconstructions were analysed. Muscle fibers originating from CFZS patients were confirmed to have undergone striking myofiber hypertrophy possessing a significantly increased CSA (cross-sectional area) approximately four-fold larger than CON (17,407 ± 9134 vs. 4046 ± 1720) (Fig. 1a and b). Furthermore, control fibers maintained a homogenous population of CSAs, whereas the CFZS patients displayed a large wide range of CSA distribution (Fig. 1c).

### CFZS patients experience myofiber force impairments

Despite the marked cellular hypertrophy observed above in 3D and previously in 2D [9, 18], CFZS patients typically maintain a normal whole muscle size. It has therefore been inferred that CFZS patients possess fewer myofibers [18]. Hence, we subsequently aimed to determine whether the overall muscle weakness present in CFZS patients was due to a reduction in force production in individual fibers rather than simply the inferred presence of hypoplasia. To assess the force generating capacity of these myofibers we measured the absolute steady state isometric force during Ca^2+^ saturation (pCa 4.5). This ensured no confounding effects of Ca^2+^ handling or sarcolemma excitability were observed.

**Fig. 2:**
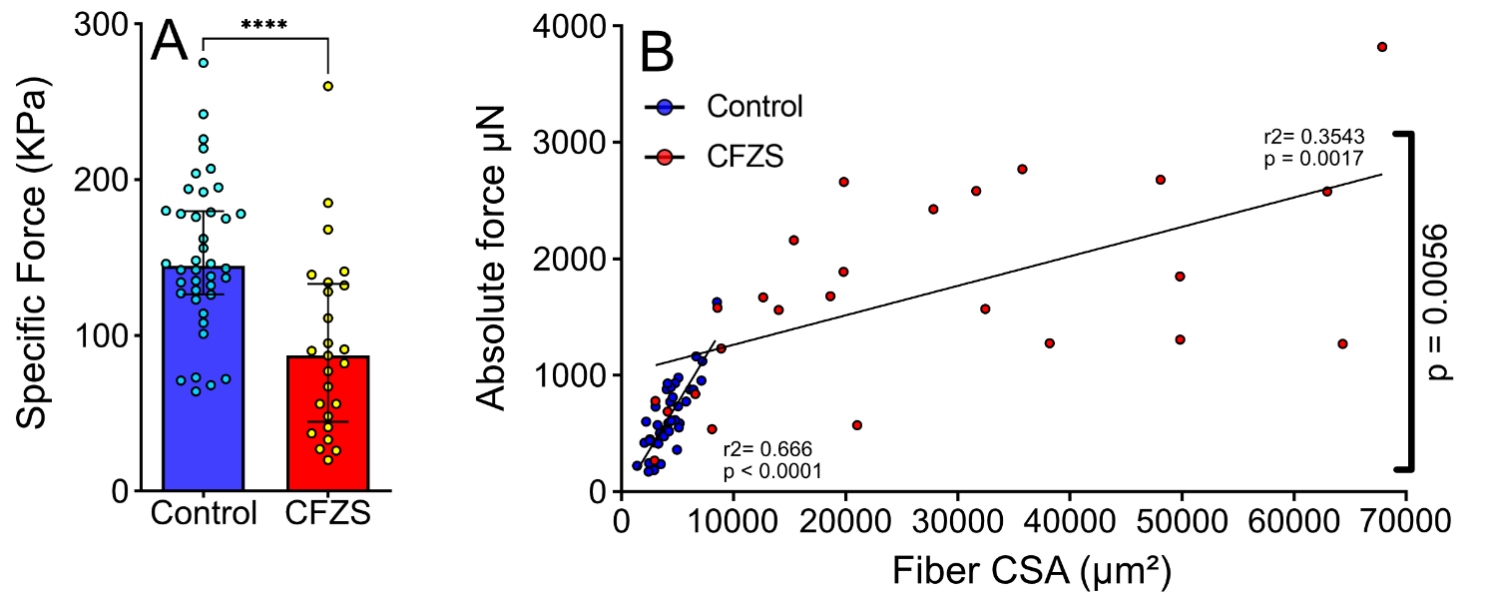
**Comparison of force production between control and CFZS patients. **(**A**) Specific force (absolute force/ CSA) for controls (N = and CFZS patients (N=, presented as median ± interquartile range. (**B**) Scatter plot depicting absolute force compared to cross-sectional area (CSA) for control (blue) and CFZS patients (red). Simple linear regression determined r^2^ values for each slope. Mann Whiney test confirmed significance between CON and CFZS for specific force, **** represents a p value of < 0.0001

While absolute force was significantly higher in CFZS patients (638.00 ± 308.10 vs. 1690 ± 8564.80, CON vs. CFZS respectively), specific force (absolute force divided by CSA) was reduced by approximately 60% (151.50 ± 48.78 vs. 93.24 ± 57.96, CON vs. CFZS respectively) (Fig. 2a). Unlike CON, absolute force was not tightly correlated to CSA in CFZS patient fibers (Fig. 2b). These findings suggest that while the larger size of the CFZS patient fibers allows an overall increase in force production, it is not as large as would be expected for a fiber of this size. Overall, these findings suggest that muscle weakness in CFZS patients is not simply due to a reduction in overall number of myofibers and that a reduced force-production capacity of individual muscle fibers is a significant confounding factor.

### CFZS patients possess an unexpected increase nuclei number, however mean MND size is enlarged and myonuclear spatial positioning are dysregulated

**Fig. 3:**
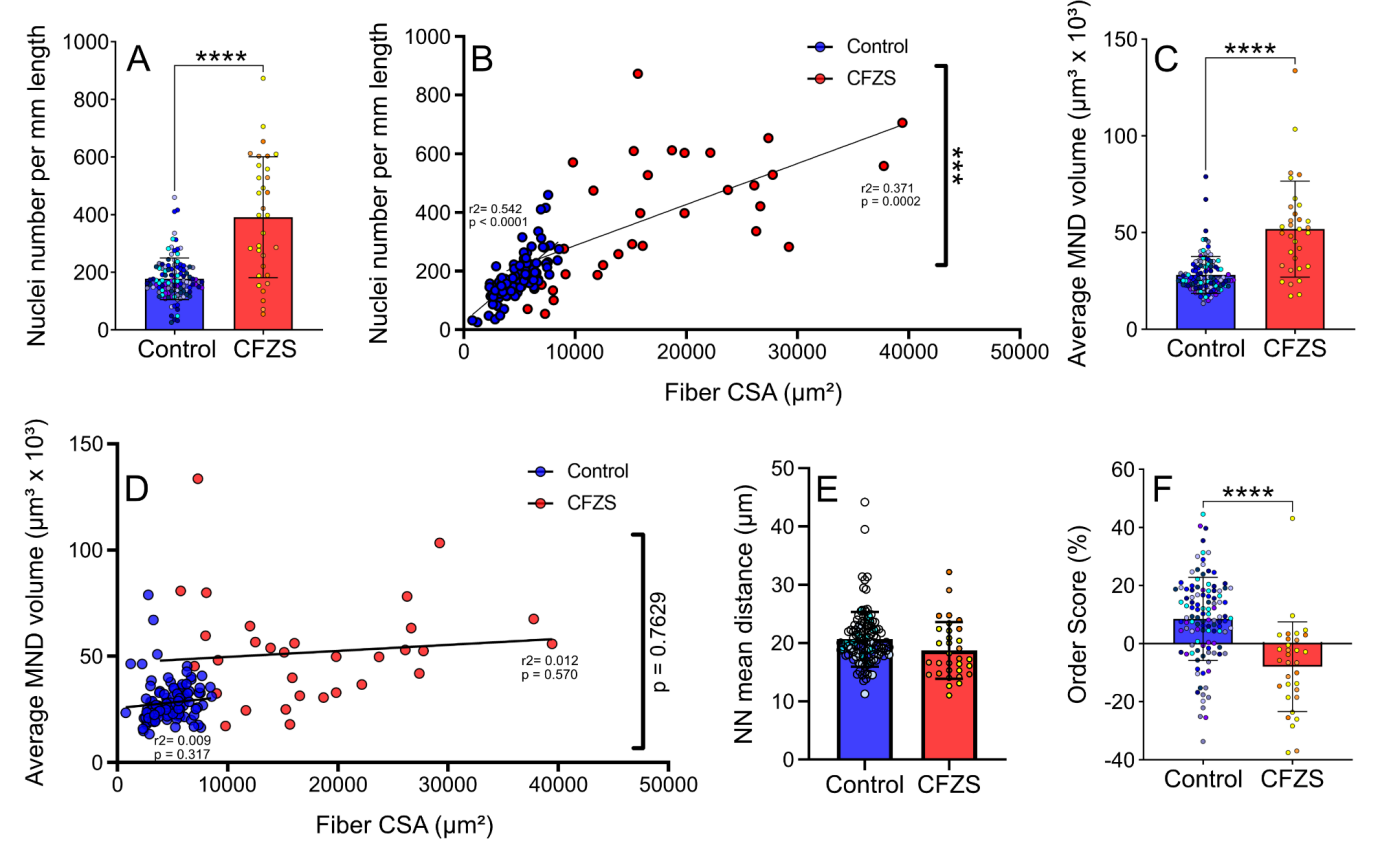
**Comparison of nuclear number and distribution.** (**A**) Nuclei number per mm length, (**B**) Scatter plot depicting nuclei number per mm length compared to cross-sectional area (CSA), (**C**) Average MND volume, (**D**) Scatter plot depicting average MND volume compared to CSA, (**E**) Nearest neighbour (NN) mean distance, (**F**) Order Score. Simple linear regression determined r^2^ values for each slope. T-test’s were utilise for all group comparisons (A, C, E and F), **** represents a p value of < 0.0001 and *** represents a p value of. All figures depict controls in blue (*N* = 114) and CFZS patients (*N* = 32) in red

As myomaker mediates fusion and therefore myonuclear accretion, we next aimed to investigate if disruptions in myonuclear number and spatial arrangement were present and may be prompting the contractile dysregulation observed in these patients. Surprisingly, we observed CFZS patients to have more than twice the number of myonuclei per mm of length, compared to CON (177.10 ± 72.18 vs. 390.80 ± 209.70, CON vs. CFZS respectively) (Fig. 3a). The CON fibers displayed a tight regulation between nuclei and CSA whereas CFZS patient fibers did not (Fig. 3b). As a result, the MND (myonuclear domain) was significantly enlarged in the CFZS patients, further indicating an inadequate nuclear number (28.10 ± 9.52 vs. 51.81 ± 24.84, CON vs. CFZS respectively (Fig. 3c and d).

As nuclei clustering was apparent in CFZS patients, (exemplified in Fig. 1a), we investigated nearest neighbour distance (NNd). Surprisingly, NNd was not significantly different between the CFZS patients and controls (Fig. 3e). To determine the distribution of nuclei along the myofiber, we then calculated the order score, whereby the higher the score the more even the distribution is. CFZS patient samples showed a significant reduction in order score which is indicative of a more random nuclei distribution compared to the controls (8.49 ± 14.30 vs. -7.95 ± 15.44, CON vs. CFZS respectively, Fig. 3f). Together, these findings indicate that while myoblast fusion is still present, myonuclei dysregulation may be prevalent and provide some insight into the impaired myofiber muscle strength.

**Fig. 4:**
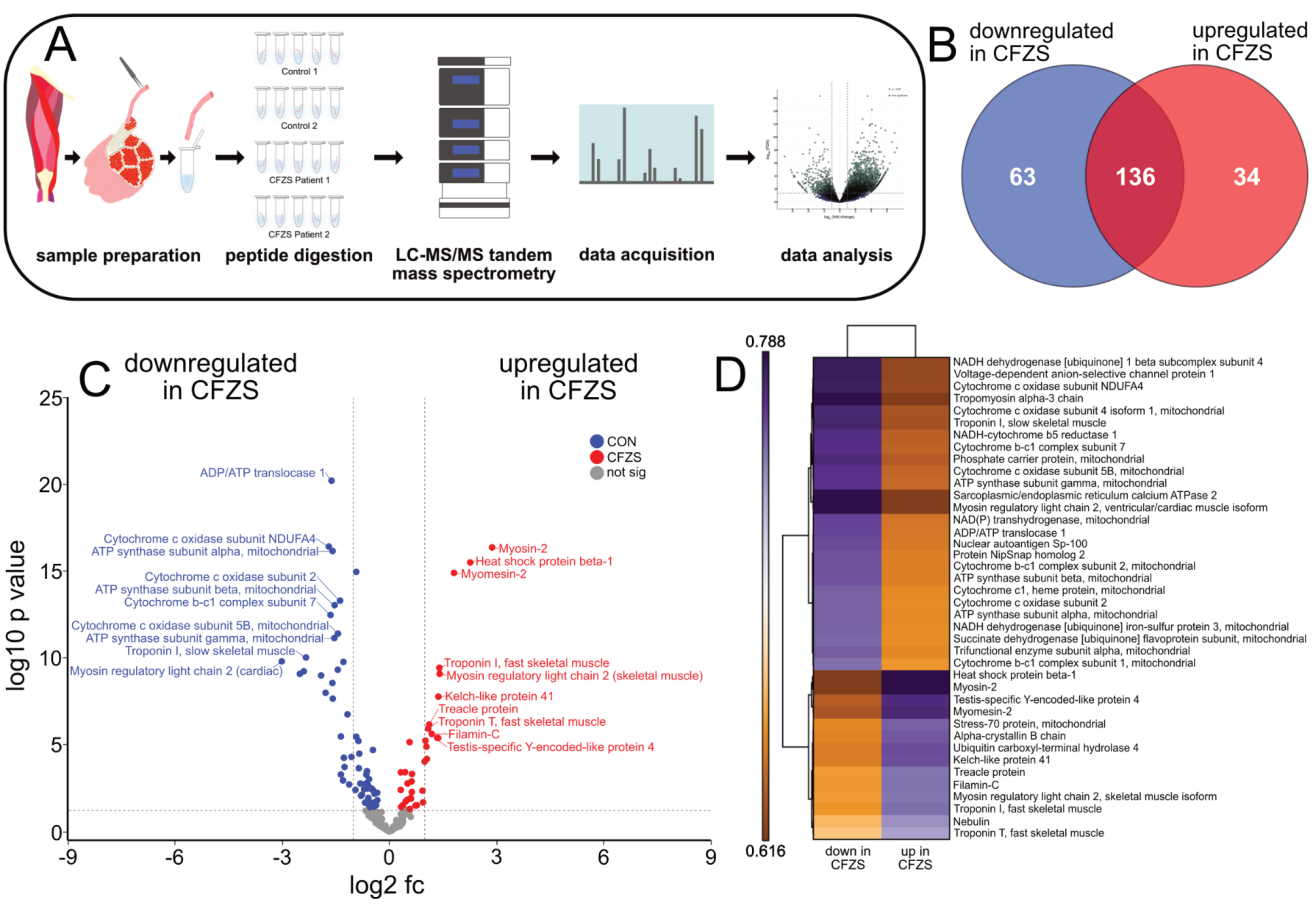
**Comparative proteomic analysis.** (**A**) Schematic depicting the process of analysing myofibers obtained from controls and CFZS patients (5 fibers collected per sample tube). (**B**) Venn diagram depicting detected proteins that were significantly upregulated and downregulated in CFZS patients as well as those that remained unchanged between experimental groups. (**C**) Volcano plot displaying Log2 fold change (Log2 fc) against Log10 *p*-value. Dark blue dots indicate *p* < 0.05 controls (aka. downregulated in CFZS) whereas red dots indicate *p* < 0.05 for CFZS patients (aka. upregulated in CFZS). Non-significant (*p* > 0.05) proteins are highlighted in grey. The top 10 most significant proteins for each experimental group have been annotated in blue (downregulated in CFZS) or red (upregulated in CFZS). (**D**) A heat map was created to illustrate the proteins with the greatest fold change, all proteins included possessed a Log2 FC > 1.5. For readability gene names rather than protein names were included in the heap map

As both the MND and the order score are dysregulated in the CFZS patients, we assumed transcriptional and subsequent proteomic alterations will be present. We therefore performed quantitative LC-MS/MS tandem mass spectrometry to gain insights into the proteomic profile present in this dysfunctional hypertrophic state (Fig. 4a). Following data filtration 233 proteins were considered for comparison between CFZS patients and control counterparts (Supplementary Table 1). Important to note, as the control group possesses the desired/ healthy proteome profile, any protein which appears upregulated in this condition are only present due to a down regulation in the CFZS group. As such, we will henceforth refer to the proteins upregulated in the control group as downregulated in the CFZS group. Of the 233 proteins considered for comparison, 97 were altered significantly between groups, with approximately 65% being downregulated in CFZS patients (63 down and 34 up CFZS, Fig. 4b). Within these 233 proteins there were no known hypertrophic proteins observed. However, we still sort to utilise this proteomic data to capture the proteomic differences present in CFZS patients. Subsequently, a volcano plot was generated to visualise differentially regulated proteins, which was annotated with the top 10 most significant proteins both up and down-regulated in CFZS patients (Fig. 4c). The protein with the most significant change and the protein with the highest fold change were both observed to be downregulated in CFZS (ADP/ATP translocase 1 and myosin regulatory light chain 2, respectively). Furthermore, all proteins with a log2fc of > 1.5 were visualised in a heatmap (Fig. 4d). This further illustrated that a greater number of proteins were significantly downregulated in CFZS. Furthermore, the heatmap also indicated that many of the differential proteins were associated with metabolic processes.

**Fig. 5:**
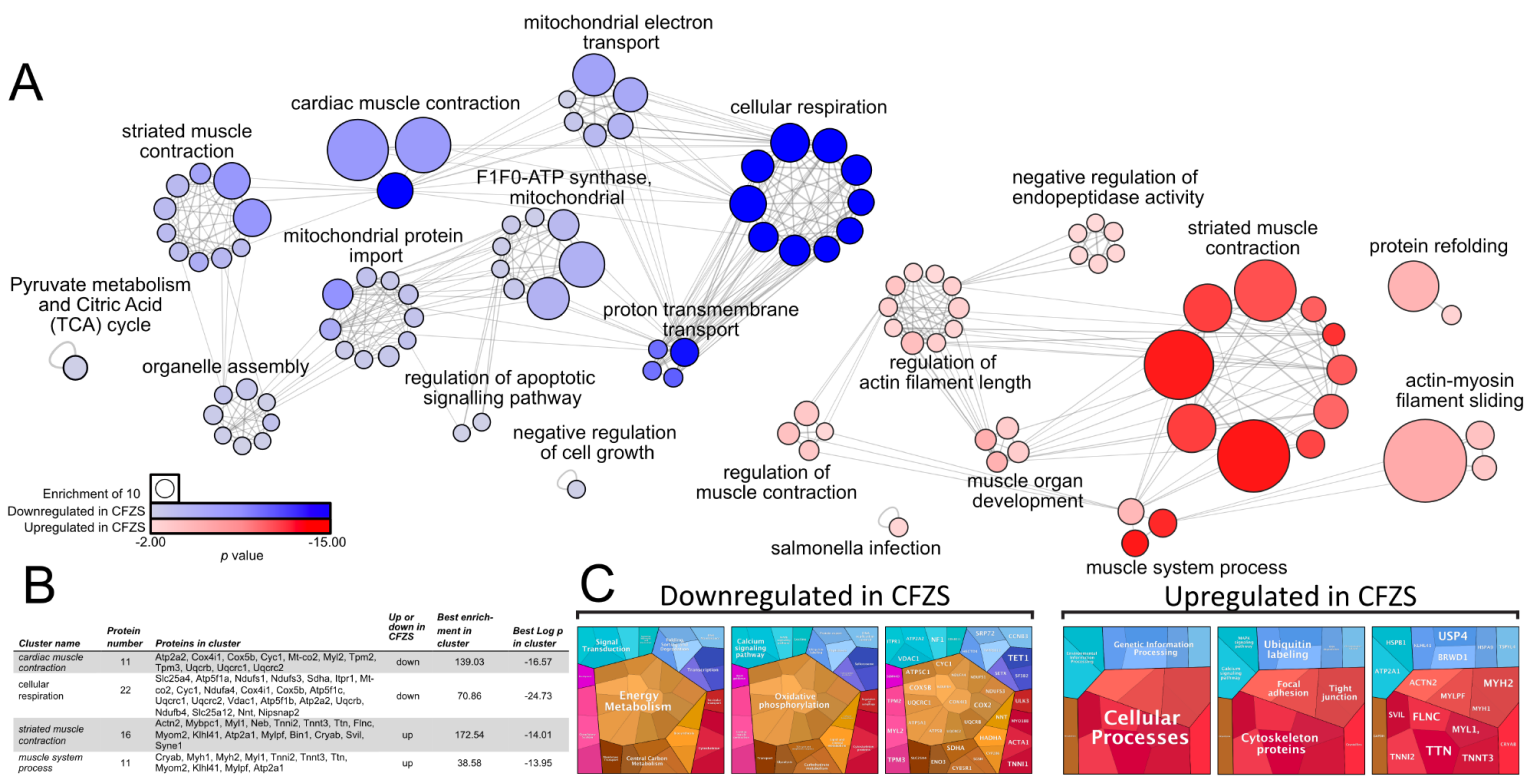
**Ontological analysis of the proteins up and down regulated in CFZS patients** (**A**) Ontological associations between 63 proteins downregulated in CFZS (blue) and 34 proteins upregulated in CFZS patients (red) were established using Metascape and visualised using Cytoscape [37, 40] Grey lines indicate a direct interaction, circle size is determined by enrichment and circle colour is determined by p value. Up and downregulated networks were created separately with identical enrichment and p value scaling parameters. For graphical representation both up and down-regulated protein networks were scaled to match based on the enrichment key. (**B**) For each experimental group the two clusters with the highest Log p in cluster are documented in the table which outlines the enrichment, p value and proteins present within the cluster. All full list of all clusters and proteins present is available in Supplementary Table 2. (**C**) Proteomaps were generated using Log2 transformed protein abundance values of proteins which displayed a significant difference (p < 0.05) [22]

To highlight functional proteomic pathways, we next performed gene ontology (GO) analysis on all proteins which passed p-value (*p* < 0.05) filtration. We first utilised the Metascape analysis resource (Fig. 5a and b, supplementary Table 2) in which the GO term ‘striated muscle contraction’, was observed in the upregulated group. Interestingly ‘striated muscle contraction’ was also downregulated in CFZS, suggesting that various proteins associated with muscle contraction are dysregulated in both directions in CFZS. It is therefore unlikely that the increase in proteins associated with straited muscle contraction is the sole contributor to the increased fiber size in CFZS patients. Further, these findings may indicate a potential compensatory hypertrophic mechanism or pathophysiological profile in dysfunctional hypertrophy.

The most significant functional group was the downregulation of cellular respiration, thus inferring a downregulated metabolic process may be present in the CFZS patients. To further examine this, we carried out additional GO analysis utilising the protein abundance values in Proteomaps. Similarly to the Metascape findings, we observed an increase in the GO term ‘cytoskeletal proteins’ to be both up and down regulated in CFZS patients, with the former displaying a greater contribution of ‘cytoskeletal proteins’ to total protein abundance than the latter (Fig. 4c). Proteomap analysis also confirmed the presence of a downregulation of metabolic associated proteins in CFZS. More specifically, there was a downregulation in protein abundance associated with oxidative phosphorylation, regardless of fiber type, (supplementary Fig. 1).

## Discussion

The pathophysiology underlying the overall impaired muscle function in the presence of striking fiber hypertrophy in patients with CFZS is not well understood. To test our hypothesis that overall muscle weakness experienced by CFZS patients is due to enlarged MND sizes and compromised intrinsic cellular force production at a single fiber level, we first confirmed the presence of hypertrophy in three-dimensions. Indeed, muscle fibers isolated from CFZS patients were approximately four-fold larger than their control counterparts. As myofiber size is typically proportional to force production [32], we therefore unsurprisingly observed an increase in absolute force capacity in the larger CFZS patient fibers. However, as demonstrated by the significantly reduced specific force, this was lower than would be expected for a fiber of this magnitude. The impaired specific force in the presence of remarkably large CSA indicates that the hypertrophy present in CFZS patients is pathological, subsequently the increased myofiber size will not compensate for their reduced number in these patients. Consequently, individual fiber force production is a contributing factor of muscle dysfunction in CFZS patients, not merely the presence of myofiber hypoplasia.

*MYMK* mutations have previously been divided into two classifications based on fusogenic capacity: null and hypomorphic [9]. Similarly to KO models, the null mutations produce no myoblast fusion [26, 39], whereas the hypomorphic mutations retain some fusogenic capacity in vitro. The CFZS patients in this study carried an as yet unclassified mutation (c.235T > C) and a mutation previously classified as hypomorphic (c.271 C > A) due to its maintenance of a control like fusogenic capacity. Surprisingly, we observe a two-fold increase in nuclear number which could be suggestive of an improved fusogenic capacity. While a decrease in myonuclear accretion has previously been observed to induce a dysfunctional phenotype in muscle disease [21] an increase in nuclear accretion has not. These findings may indicate the importance of nuclear accretion being tightly regulated in either direction (increase or decrease) as functional impairments may be induced following any deviation from the optimal fiber requirements.

Although nuclei number is increased two-fold in the CFZS patient fibers the overall size is approximately four-fold higher, highlighting that the observed increase in myonuclear number may not be sufficient to support optimal function in a myofiber of this magnitude and may subsequently be indicative of a myonuclear impairment. The significant increase in MND size further supports the concept of impaired myonuclear accretion in CFZS patients, whereby this enlarged MND size is typically induces impaired force production [32]. Indeed, myoblasts cultured from a patient with the same hypomorphic mutation as those in the current study produced both an increase in mono-nucleated myofibers and a decrease in multi-nucleated fibers, which is also indictive of a fusogenic impairment [9]. Interestingly, while these patient derived myoblasts suggested a fusion impairment, myoblasts modified to carry only the patient’s hypomorphic mutation, recorded a maintenance in fusion capacity. Fusogenic capacity of each *MYMK* mutation in isolation may therefore not be indicative of a patients pathophenotype due to the presence of a second mutation. Furthermore, the impaired fusion observed in patient myotubes in vitro also suggest a reduction in myotube diameter which does not recapitulate their accompanying histological analysis. The varied pathophenotype between in vivo and in vitro derived myofibers from CFZS patients may therefore indicate a preference or potential mechanistic difference between fusion required for production of new fibers compared to fusion requirements for existing fibers (myonuclear accretion). This may partially explain the reduction in total number of myofibers but an increase in nuclear number observed in the patient derived single fibers in the current study.

As with reduced nuclei number, aberrant myonuclear arrangement has previously been observed to impair myotube function in muscle disease [35]. Indeed, the regulation of nuclei location was impaired within CFZS patients (as determined by the order score), thus diminished transport of essential mRNA and protein across the entire myofiber is a likely driver of the impaired myofibril contractility observed [3, 17]. These findings indicate that *MYMK* mutations not only affect fusion capacity but may also play a distinct role in nuclei location once myonuclear accretion has taken place [32].

To provide insight into the striking myofiber hypertrophy observed in these individuals we performed proteomics on myofibers, derived from both patients and controls. Unfortunately, we did not uncover proteins with a known hypertrophic association, however this may be due to the temporal regulation of hypertrophy in skeletal muscle [2, 7]. To fully establish these mechanisms a model of muscle damage/ growth in conjunction with CFZS causing mutations would therefore be required. We did not observe the presence of myomaker in either the patient or control cohorts. This is not unsurprising as these fibers were under homeostatic balance and the requirement for nuclear accretion was likely low. This finding also suggests that the muscle dysfunction observed in these patients is likely not associated with impaired membrane integrity, as previously observed in dystrophic mice when myomaker is present outside of fusogenic requirements [31]. One limitation of the methodology utilised here however, is that sarcomeric proteins are highly abundant, which may consequently lead to less abundant proteins such as transcription factors and known secretory proteins being below the limit of detection of this analysis, therefore further confirmation of this finding is required. CFZS patients possessed a significant dysregulation of proteins associated with striated muscle contraction/ cytoskeleton (different proteins associated with this functional group were both up and down regulated). Changes in contractile proteins is likely to contribute to the impaired force production and striking myofiber hypertrophy previously observed in the CFZS patients. While these findings may indicate a possible pathological contractile profile or indeed the presence of a compensatory mechanisms we were unable to ascertain the influence of this proteomic profile in relation to the force dysfunction. Mechanistic understanding should therefore be addressed in future studies.

Proteomic profiling also allowed for the observation that a decrease in proteins associated with cellular respiration and more specifically, oxidative phosphorylation was present in CFZS patient fibers. Unfortunately, these findings do not indicate a causal relationship, however previous identification of pathways associated with increased aerobic respiration elucidated an apparent fiber type switching or reliance on slow fiber types have been observed in another muscular disorder [34]. It may therefore be reasonable to suggest the reverse could also be true; whereby a reduction in the capacity for oxidative phosphorylation (as is the case in CFZS patients here) may increase reliance or be indicative of a partial switch towards fast twitch fibers (which possess greater hypertrophic capacity). Although not present in this cohort [18], this may be one justification for fast fibers being reported to selectively undergo hypertrophy in some CFZS patients [9]. No further mechanistic studies were undertaken within the scope of this study. Future investigations should therefore focus on whether these apparent metabolic differences induce functional and structural changes. This should include electron microscopic analysis to provide ultrastructural assessment of CFZS muscle, with a particular focus on the abundance and structure of mitochondria and myofibrils. Furthermore, determination of mitochondrial respiratory capacity in these patients should be prioritised, to determine if this proteomic profile affects function. Together with our findings, these future studies may provide an indication of whether theses metabolic differences could present as a druggable target to improve functional outputs in these patients in future.

## Conclusions

In summary, we have demonstrated that CFZS patients possess dysfunctional hypertrophic myofibers, which are unlikely to compensate for reduced number of fibers. We suggest this dysfunction is due in part to dysregulated MND size and a dysregulated protein profile including a perturbation in the regulation of oxidative phosphorylation proteins.

### Electronic supplementary material

Below is the link to the electronic supplementary material.

Supplementary Material 1

## Data Availability

All the raw data can be accessed on request.

## References

[1] Alrohaif H, Topf A, Evangelista T, Lek M, McArthur D, Lochmuller H (2018). Whole-exome sequencing identifies mutations in MYMK in a mild form of Carey-Fineman-Ziter syndrome. Neurol Genet.

[2] Brook MS, Wilkinson DJ, Smith K, Atherton PJ (2016). The metabolic and temporal basis of muscle hypertrophy in response to resistance exercise. Eur J Sport Sci.

[3] Bruusgaard J, Liestøl K, Ekmark M, Kollstad K, Gundersen K (2003). Number and spatial distribution of nuclei in the muscle fibres of normal mice studied in vivo. J Physiol.

[4] Camacho A, Martinez B, Alvarez S, Gil-Fournier B, Ramiro S, Hernandez-Lain A, Nunez N, Simon R (2020). Carey-Fineman-Ziter syndrome: a MYMK-Related Myopathy Mimicking Brainstem Dysgenesis. J Neuromuscul Dis.

[5] Carey JC (2004). The Carey-Fineman-Ziter syndrome: follow-up of the original siblings and comments on pathogenesis. Am J Med Genet A.

[6] Carey JC, Fineman RM, Ziter FA (1982). The Robin sequence as a consequence of malformation, dysplasia, and neuromuscular syndromes. J Pediatr.

[7] Chaillou T, Lee JD, England JH, Esser KA, McCarthy JJ (2013). Time course of gene expression during mouse skeletal muscle hypertrophy. J Appl Physiol.

[8] Cheek DB, Holt AB, Hill DE, Talbert JL (1971). Skeletal muscle cell mass and growth: the concept of the deoxyribonucleic acid unit. Pediatr Res.

[9] Di Gioia SA, Connors S, Matsunami N, Cannavino J, Rose MF, Gilette NM, Artoni P, de Macena Sobreira NL, Chan WM, Webb BD et al (2017) A defect in myoblast fusion underlies Carey-Fineman-Ziter syndrome. Nat Commun 8: 16077 10.1038/ncomms16077 10.1038/ncomms16077 PMC5504296 28681861

[10] Eng JK, McCormack AL, Yates JR (1994). An approach to correlate tandem mass spectral data of peptides with amino acid sequences in a protein database. J Am Soc Mass Spectrom.

[11] Epstein CJ (1967). Cell size, nuclear content, and the development of polyploidy in the mammalian liver. Proc Natl Acad Sci U S A.

[12] Frontera WR, Larsson L (1997). Contractile studies of single human skeletal muscle fibers: a comparison of different muscles, permeabilization procedures, and storage techniques. Muscle Nerve.

[13] Frontera WR, Ochala J (2015). Skeletal muscle: a brief review of structure and function. Calcif Tissue Int.

[14] Goh Q, Millay DP (2017) Requirement of myomaker-mediated stem cell fusion for skeletal muscle hypertrophy. Elife 6. 10.7554/eLife.20007 10.7554/eLife.20007 PMC5338923 28186492

[15] Goh Q, Song T, Petrany MJ, Cramer AA, Sun C, Sadayappan S, Lee S-J, Millay DP (2019). Myonuclear accretion is a determinant of exercise-induced remodeling in skeletal muscle. Elife.

[16] Hall ZW, Ralston E (1989). Nuclear domains in muscle cells. Cell.

[17] Hansson K-A, Solbrå AV, Gundersen K, Bruusgaard JC (2020). Computational Assessment of Transport Distances in Living Skeletal Muscle Fibers studied in situ. Biophys J.

[18] Hedberg-Oldfors C, Lindberg C, Oldfors A (2018). Carey-Fineman-Ziter syndrome with mutations in the myomaker gene and muscle fiber hypertrophy. Neurol Genet.

[19] Kingma DW, Feeback DL, Marks WA, Bobele GB, Leech RW, Brumback RA (1991). Selective type II muscle Fiber hypertrophy in severe infantile spinal muscular atrophy. J Child Neurol.

[20] Kornegay JN, Childers MK, Bogan DJ, Bogan JR, Nghiem P, Wang J, Fan Z, Howard JF Jr., Schatzberg SJ, Dow JL (2012) al The paradox of muscle hypertrophy in muscular dystrophy. Phys Med Rehabil Clin N Am 23: 149–172, xii 10.1016/j.pmr.2011.11.014 10.1016/j.pmr.2011.11.014 PMC5951392 22239881

[21] Levy Y, Ross JA, Niglas M, Snetkov VA, Lynham S, Liao CY, Puckelwartz MJ, Hsu YM, Alsheimer McNally EM M et al (2018) Prelamin A causes aberrant myonuclear arrangement and results in muscle fiber weakness. JCI Insight 3: 10.1172/jci.insight.120920 10.1172/jci.insight.120920 PMC6237469 30282816

[22] Liebermeister W, Noor E, Flamholz A, Davidi D, Bernhardt J, Milo R (2014). Visual account of protein investment in cellular functions. Proc Natl Acad Sci U S A.

[23] Lindqvist J, Cheng AJ, Renaud G, Hardeman EC, Ochala J (2013). Distinct underlying mechanisms of limb and respiratory muscle fiber weaknesses in nemaline myopathy. J Neuropathol Exp Neurol.

[24] Lindqvist J, Levy Y, Pati-Alam A, Hardeman EC, Gregorevic P, Ochala J (2016). Modulating myosin restores muscle function in a mouse model of nemaline myopathy. Ann Neurol.

[25] Luo W, Li E, Nie Q, Zhang X (2015). Myomaker, regulated by MYOD, MYOG and miR-140-3p, promotes Chicken Myoblast Fusion. Int J Mol Sci.

[26] Millay DP, O’Rourke JR, Sutherland LB, Bezprozvannaya S, Shelton JM, Bassel-Duby R, Olson EN (2013). Myomaker is a membrane activator of myoblast fusion and muscle formation. Nature.

[27] Millay DP, Sutherland LB, Bassel-Duby R, Olson EN (2014). Myomaker is essential for muscle regeneration. Genes Dev.

[28] Murgia M, Toniolo L, Nagaraj N, Ciciliot S, Vindigni V, Schiaffino S, Reggiani C, Mann M (2017). Single muscle Fiber Proteomics reveals Fiber-type-specific features of human muscle aging. Cell Rep.

[29] Ochala J, Ravenscroft G, Laing NG, Nowak KJ (2012). Nemaline myopathy-related skeletal muscle α-actin (ACTA1) mutation, Asp286Gly, prevents proper strong myosin binding and triggers muscle weakness. PLoS ONE.

[30] Pavlath GK, Rich K, Webster SG, Blau HM (1989). Localization of muscle gene products in nuclear domains. Nature.

[31] Petrany MJ, Song T, Sadayappan S, Millay DP (2020) Myocyte-derived myomaker expression is required for regenerative fusion but exacerbates membrane instability in dystrophic myofibers. JCI insight. 10.1172/jci.insight.136095 10.1172/jci.insight.136095 PMC7253022 32310830

[32] Qaisar R, Renaud G, Morine K, Barton ER, Sweeney HL, Larsson L (2012). Is functional hypertrophy and specific force coupled with the addition of myonuclei at the single muscle fiber level?. Faseb j.

[33] Ramirez-Martinez A, Zhang Y, van den Boogaard MJ, McAnally JR, Rodriguez-Caycedo C, Chai AC, Chemello F, Massink MP, Cuppen I, Elferink MG et al (2022) Impaired activity of the fusogenic micropeptide Myomixer causes myopathy resembling Carey-Fineman-Ziter syndrome. J Clin Invest 132: 10.1172/jci159002 10.1172/JCI159002 PMC9151691 35642635

[34] Ranu N, Laitila J, Dugdale HF, Mariano J, Kolb JS, Wallgren-Pettersson C, Witting N, Vissing J, Vilchez JJ, Fiorillo Cet al (2022). NEB mutations disrupt the super-relaxed state of myosin and remodel the muscle metabolic proteome in nemaline myopathy. Acta Neuropathol Commun.

[35] Ross JA, Tasfaout H, Levy Y, Morgan J, Cowling BS, Laporte J, Zanoteli E, Romero NB, Lowe DA Jungbluth H (2020) rAAV-related therapy fully rescues myonuclear and myofilament function in X-linked myotubular myopathy. Acta Neuropathol Commun 8: 167 10.1186/s40478-020-01048-8 10.1186/s40478-020-01048-8 PMC7574461 33076971

[36] Ross JA, Levy Y, Ripolone M, Kolb JS, Turmaine M, Holt M, Lindqvist J, Claeys KG, Weis J Monforte M (2019) impairments in contractility and cytoskeletal organisation cause nuclear defects in nemaline myopathy. Acta Neuropathol 138: 477–495 10.1007/s00401-019-02034-8 10.1007/s00401-019-02034-8 PMC6689292 31218456

[37] Shannon P, Markiel A, Ozier O, Baliga NS, Wang JT, Ramage D, Amin N, Schwikowski B, Ideker T (2003). Cytoscape: a software environment for integrated models of biomolecular interaction networks. Genome Res.

[38] Windner SE, Manhart A, Brown A, Mogilner A, Baylies MK (2019). Nuclear scaling is coordinated among individual nuclei in multinucleated muscle fibers. Dev Cell.

[39] Zhang W, Roy S (2017). Myomaker is required for the fusion of fast-twitch myocytes in the zebrafish embryo. Dev Biol.

[40] Zhou Y, Zhou B, Pache L, Chang M, Khodabakhshi AH, Tanaseichuk O, Benner C, Chanda SK (2019). Metascape provides a biologist-oriented resource for the analysis of systems-level datasets. Nat Commun.

