# Supplementary material for: Aberrant myonuclear domains and impaired myofiber contractility despite marked hypertrophy in MYMK-related, Carey-Fineman-Ziter Syndrome
Source: Acta Neuropathol Commun. 2024 May 24;12:80. doi: 10.1186/s40478-024-01783-2 (PMC11127446; doi:10.1186/s40478-024-01783-2)
Supplement: Supplementary file 1 — Supplementary Material 1 [file 40478_2024_1783_MOESM1_ESM.docx]

**Aberrant myonuclear domains and myofiber contractility despite marked hypertrophy in *MYMK*-related, Carey Fineman Ziter Syndrome.**

Supplementary figure 1

**Fiber type divided comparisons between controls and patients fibers.** All control fibers were determined to be mixed/ slow fibers, whereas half the patient fibers were mixed/ slow and the rest were fast type 2A fibers. Both the PCA plot (A) and the pearson correlation comparisons (B) display distinct differences between control and patients despite fiber type, further (C) depicts each protein associated as involvement in cellular respiration following metascape analysis with statistical differences obtained from one-way ANOVA using average abundance, significance is determined as p ≤ 0.05 (p ≤ 0.0001 = ****, p ≤ 0.001 = ***, p ≤ 0.01 = ** and p ≤ 0.05 = *).


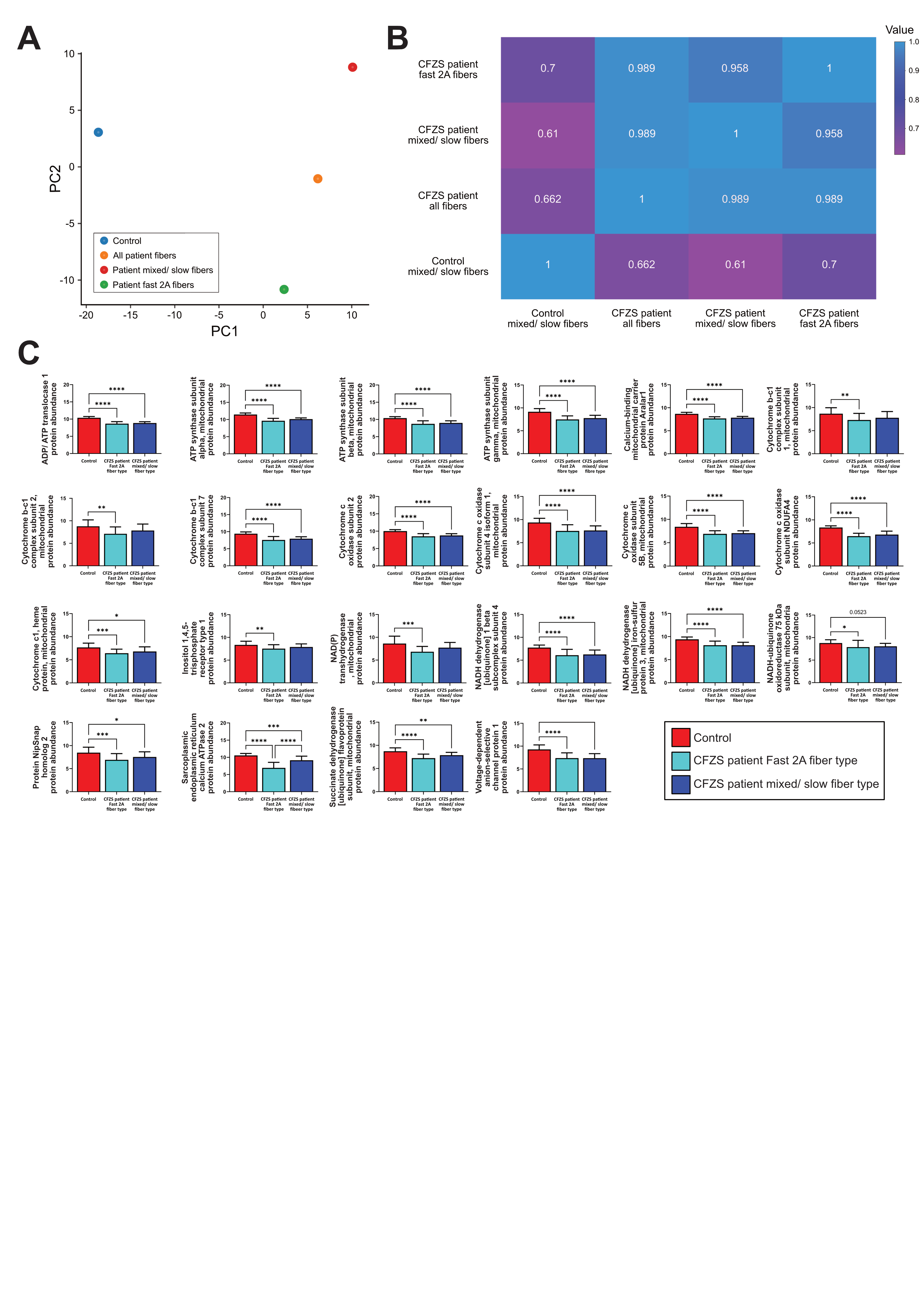


Supplementary table 1

**All proteins detected in manually dissected fibers originating from heathy controls and CFZS patients**

All proteins detected during LC-MS/MS tandem mass spectrometry, following filtration of missing values. #protein abundance values. Significant upregulation in each experimental group determined based on p < 0.05.

| ***Gene name*** | ***Uniprot ID*** | ***Protein name*** | ***Healthy control ^#^*** | ***CFZS ^#^*** | ***Log_2_ FC*** | ***p value*** | ***q value*** |
| --- | --- | --- | --- | --- | --- | --- | --- |
| **UPREGULATED IN CFZS PATIENTS** | | | | | | | |
| MYH2 | Q9UKX2 | Myosin-2 |  |  | 2.878 | <0.001 | <0.001 |
| HSPB1 | P04792 | Heat shock protein beta-1 |  |  | 2.266 | <0.001 | <0.001 |
| MYOM2 | P54296 | Myomesin-2 |  |  | 1.812 | <0.001 | <0.001 |
| MYLPF | Q96A32 | Myosin regulatory light chain 2, skeletal muscle isoform |  |  | 1.410 | <0.001 | <0.001 |
| TNNI2 | P48788 | Troponin I, fast skeletal muscle |  |  | 1.403 | <0.001 | <0.001 |
| KLHL41 | O60662 | Kelch-like protein 41 |  |  | 1.375 | <0.001 | <0.001 |
| CRYAB | P02511 | Alpha-crystallin B chain |  |  | 1.368 | <0.001 | <0.001 |
| TSPYL4 | Q9UJ04 | Testis-specific Y-encoded-like protein 4 |  |  | 1.344 | <0.001 | <0.001 |
| FLNC | Q14315 | Filamin-C |  |  | 1.190 | <0.001 | <0.001 |
| TCOF1 | Q13428 | Treacle protein |  |  | 1.119 | <0.001 | <0.001 |
| TNNT3 | P45378 | Troponin T, fast skeletal muscle |  |  | 1.090 | <0.001 | <0.001 |
| NEB | P20929 | Nebulin |  |  | 1.054 | <0.001 | <0.001 |
| USP4 | Q13107 | Ubiquitin carboxyl-terminal hydrolase 4 |  |  | 1.040 | <0.001 | <0.001 |
| HSPA9 | P38646 | Stress-70 protein, mitochondrial |  |  | 1.012 | <0.001 | <0.001 |
| GPRIN1 | Q7Z2K8 | G protein-regulated inducer of neurite outgrowth 1 |  |  | 0.990 | <0.001 | <0.001 |
| DLG5 | Q8TDM6 | Disks large homolog 5 |  |  | 0.947 | 0.021 | 0.044 |
| SVIL | O95425 | Supervillin |  |  | 0.930 | 0.004 | 0.011 |
| SETBP1 | Q9Y6X0 | SET-binding protein |  |  | 0.771 | 0.031 | 0.062 |
| MYH1 | P12882 | Myosin-1 |  |  | 0.738 | 0.032 | 0.063 |
| TTN | Q8WZ42 | Titin |  |  | 0.637 | <0.001 | 0.002 |
| BRWD1 | Q9NSI6 | Bromodomain and WD repeat-containing protein 1 |  |  | 0.631 | 0.005 | 0.013 |
| SMCHD1 | A6NHR9 | Structural maintenance of chromosomes flexible hinge domain-containing protein 1 |  |  | 0.626 | 0.001 | 0.004 |
| C10orf120 | Q5SQS8 | Uncharacterized protein C10orf120 |  |  | 0.608 | 0.013 | 0.028 |
| HIRIP3 | Q9BW71 | HIRA-interacting protein 3 |  |  | 0.602 | 0.011 | 0.027 |
| CCDC7 | Q96M83 | Coiled-coil domain-containing protein 7 |  |  | 0.577 | 0.050 | 0.099 |
| ACTN2 | P35609 | Alpha-actinin-2 |  |  | 0.568 | <0.001 | <0.001 |
| GAPDH | P04406 | Glyceraldehyde-3-phosphate dehydrogenase |  |  | 0.515 | 0.014 | 0.032 |
| MYL1 | P05976 | Myosin light chain 1/3, skeletal muscle isoform |  |  | 0.513 | 0.002 | 0.005 |
| BIN1 | O00499 | Myc box-dependent-interacting protein 1 |  |  | 0.458 | 0.018 | 0.040 |
| SYNE1 | Q8NF91 | Nesprin-1 |  |  | 0.444 | <0.001 | 0.001 |
| TCF20 | Q9UGU0 | Transcription factor 20 |  |  | 0.380 | 0.030 | 0.062 |
| PLCL1 | Q15111 | Inactive phospholipase C-like protein 1 |  |  | 0.330 | 0.038 | 0.076 |
| MYBPC1 | Q00872 | Myosin-binding protein C, slow-type |  |  | 0.324 | <0.001 | 0.001 |
| ATP2A1 | O14983 | Sarcoplasmic/endoplasmic reticulum calcium ATPase 1 |  |  | 0.321 | 0.004 | 0.010 |
|  |  |  |  |  |  |  |  |
| **DOWNREGULATED IN CFZS PATIENTS (UPREGULATED IN HEALTHY CONTROLS)** | | | | | | | |
|  |  |  |  |  |  |  |  |
| ASPM | Q8IZT6 | Abnormal spindle-like microcephaly-associated protein |  |  | -0.677 | 0.021 | 0.045 |
| ACTA1 | P68133 | Actin, alpha skeletal muscle |  |  | -0.457 | <0.001 | <0.001 |
| SLC25A4 | P12235 | ADP/ATP translocase 1 |  |  | -1.615 | <0.001 | <0.001 |
| ATP5F1A | P25705 | ATP synthase subunit alpha, mitochondrial |  |  | -1.585 | <0.001 | <0.001 |
| ATP5F1B | P06576 | ATP synthase subunit beta, mitochondrial |  |  | -1.528 | <0.001 | <0.001 |
| ATP5F1C | P36542 | ATP synthase subunit gamma, mitochondrial |  |  | -1.538 | <0.001 | <0.001 |
| ENO3 | P13929 | Beta-enolase |  |  | -0.413 | 0.007 | 0.016 |
| BAZ2A | Q9UIF9 | Bromodomain adjacent to zinc finger domain protein 2A |  |  | -0.863 | <0.001 | <0.001 |
| SLC25A12 | O75746 | Calcium-binding mitochondrial carrier protein Aralar1 |  |  | -0.924 | <0.001 | <0.001 |
| CEP135 | Q66GS9 | Centrosomal protein of 135 kDa |  |  | -0.790 | 0.009 | 0.021 |
| COLEC11 | Q9BWP8 | Collectin-11 |  |  | -0.852 | <0.001 | 0.001 |
| UQCRC1 | P31930 | Cytochrome b-c1 complex subunit 1, mitochondrial |  |  | -1.122 | 0.002 | 0.006 |
| UQCRC2 | P22695 | Cytochrome b-c1 complex subunit 2, mitochondrial |  |  | -1.294 | 0.001 | 0.004 |
| UQCRB | P14927 | Cytochrome b-c1 complex subunit 7 |  |  | -1.645 | <0.001 | <0.001 |
| MT-CO2 | P00403 | Cytochrome c oxidase subunit 2 |  |  | -1.376 | <0.001 | <0.001 |
| COX4I1 | P13073 | Cytochrome c oxidase subunit 4 isoform 1, mitochondrial |  |  | -1.782 | <0.001 | <0.001 |
| COX5B | P10606 | Cytochrome c oxidase subunit 5B, mitochondrial |  |  | -1.437 | <0.001 | <0.001 |
| NDUFA4 | O00483 | Cytochrome c oxidase subunit NDUFA4 |  |  | -1.691 | <0.001 | <0.001 |
| CYC1 | P08574 | Cytochrome c1, heme protein, mitochondrial |  |  | -1.058 | <0.001 | <0.001 |
| CYP2B6 | P20813 | Cytochrome P450 2B6 |  |  | -0.566 | 0.001 | 0.003 |
| DNAAF1 | Q8NEP3 | Dynein assembly factor 1, axonemal |  |  | -0.765 | 0.007 | 0.018 |
| HECTD1 | Q9ULT8 | E3 ubiquitin-protein ligase HECTD1 |  |  | -0.543 | 0.039 | 0.076 |
| CCNB3 | Q8WWL7 | G2/mitotic-specific cyclin-B3 |  |  | -0.946 | 0.004 | 0.010 |
| HEATR1 | Q9H583 | HEAT repeat-containing protein 1 |  |  | -0.592 | 0.002 | 0.006 |
| ITPR1 | Q14643 | Inositol 1,4,5-trisphosphate receptor type 1 |  |  | -0.644 | 0.003 | 0.007 |
| LRRC27 | Q9C0I9 | Leucine-rich repeat-containing protein 27 |  |  | -0.644 | 0.001 | 0.002 |
| NIPAL4 | Q0D2K0 | Magnesium transporter NIPA4 |  |  | -0.462 | 0.027 | 0.056 |
| TET1 | Q8NFU7 | Methylcytosine dioxygenase TET1 |  |  | -0.370 | 0.030 | 0.062 |
| MYL2 | P10916 | Myosin regulatory light chain 2, ventricular/cardiac muscle isoform |  |  | -3.010 | <0.001 | <0.001 |
| NNT | Q13423 | NAD(P) transhydrogenase, mitochondrial |  |  | -1.354 | 0.001 | 0.002 |
| NDUFB4 | O95168 | NADH dehydrogenase [ubiquinone] 1 beta subcomplex subunit 4 |  |  | -1.591 | <0.001 | <0.001 |
| NDUFS3 | O75489 | NADH dehydrogenase [ubiquinone] iron-sulfur protein 3, mitochondrial |  |  | -1.282 | <0.001 | <0.001 |
| CYB5R1 | Q9UHQ9 | NADH-cytochrome b5 reductase 1 |  |  | -1.355 | <0.001 | <0.001 |
| NDUFS1 | P28331 | NADH-ubiquinone oxidoreductase 75 kDa subunit, mitochondrial |  |  | -0.809 | 0.002 | 0.005 |
| NF1 | P21359 | Neurofibromin |  |  | -0.489 | 0.003 | 0.009 |
| SGSH | P51688 | N-sulphoglucosamine sulphohydrolase |  |  | -0.333 | 0.016 | 0.034 |
| SP100 | P23497 | Nuclear autoantigen Sp-100 |  |  | -1.270 | 0.000 | 0.000 |
| NUMA1 | Q14980 | Nuclear mitotic apparatus protein 1 |  |  | -0.588 | 0.019 | 0.041 |
| RP1 | P56715 | Oxygen-regulated protein 1 |  |  | -0.556 | 0.003 | 0.009 |
| PDLIM5 | Q96HC4 | PDZ and LIM domain protein 5 |  |  | -0.457 | 0.022 | 0.046 |
| SLC25A3 | Q00325 | Phosphate carrier protein, mitochondrial |  |  | -1.581 | <0.001 | <0.001 |
| SETX | Q7Z333 | Probable helicase senataxin |  |  | -0.702 | 0.004 | 0.009 |
| NIPSNAP2 | O75323 | Protein NipSnap homolog 2 |  |  | -1.252 | <0.001 | 0.001 |
| ATP2A2 | P16615 | Sarcoplasmic/endoplasmic reticulum calcium ATPase 2 |  |  | -2.503 | <0.001 | <0.001 |
| SEMA4D | Q92854 | Semaphorin-4D |  |  | -0.422 | 0.035 | 0.070 |
| ULK3 | Q6PHR2 | Serine/threonine-protein kinase ULK3 |  |  | -0.839 | <0.001 | <0.001 |
| ANKRD52 | Q8NB46 | Serine/threonine-protein phosphatase 6 regulatory ankyrin repeat subunit C |  |  | -0.606 | 0.003 | 0.009 |
| SRP72 | O76094 | Signal recognition particle subunit SRP72 |  |  | -0.603 | 0.003 | 0.008 |
| SF3B2 | Q13435 | Splicing factor 3B subunit 2 |  |  | -0.658 | 0.002 | 0.005 |
| SAMD9L | Q8IVG5 | Sterile alpha motif domain-containing protein 9-like |  |  | -0.456 | 0.004 | 0.010 |
| SDHA | P31040 | Succinate dehydrogenase [ubiquinone] flavoprotein subunit, mitochondrial |  |  | -1.170 | <0.001 | <0.001 |
| TTC28 | Q96AY4 | Tetratricopeptide repeat protein 28 |  |  | -0.410 | 0.005 | 0.013 |
| TMPRSS13 | Q9BYE2 | Transmembrane protease serine 13 |  |  | -0.560 | 0.020 | 0.044 |
| HADHA | P40939 | Trifunctional enzyme subunit alpha, mitochondrial |  |  | -1.441 | <0.001 | <0.001 |
| TPM3 | P06753 | Tropomyosin alpha-3 chain |  |  | -2.393 | <0.001 | <0.001 |
| TPM2 | P07951 | Tropomyosin beta chain |  |  | -0.337 | 0.006 | 0.014 |
| TNNI1 | P19237 | Troponin I, slow skeletal muscle |  |  | -2.330 | <0.001 | <0.001 |
| KIAA1109 | Q2LD37 | Uncharacterized protein KIAA1109 |  |  | -0.414 | 0.028 | 0.058 |
| MYO18B | Q8IUG5 | Unconventional myosin-XVIIIb |  |  | -0.622 | <0.001 | 0.001 |
| C4orf47 | A7E2U8 | UPF0602 protein C4orf47 |  |  | -0.924 | <0.001 | <0.001 |
| VDAC1 | P21796 | Voltage-dependent anion-selective channel protein 1 |  |  | -1.908 | <0.001 | <0.001 |
| WDR87 | Q6ZQQ6 | WD repeat-containing protein 87 |  |  | -0.585 | 0.012 | 0.026 |
| ZNF469 | Q96JG9 | Zinc finger protein 469 |  |  | -0.560 | 0.031 | 0.063 |
|  |  |  |  |  |  |  |  |
| **NO SIGNIFICANT DIFFERENCES BETWEEN HEALTHY CONTROLS AND CFZS PATIENTS** | | | | | | | |
| TRPM2 | O94759 | Transient receptor potential cation channel subfamily M member 2 |  |  | -0.495 | 0.052 | 0.102 |
| TTC3 | P53804 | E3 ubiquitin-protein ligase TTC3 |  |  | 0.428 | 0.054 | 0.106 |
| KIAA1107 | Q9UPP5 | Uncharacterized protein KIAA1107 |  |  | 0.509 | 0.055 | 0.107 |
| MB | P02144 | Myoglobin |  |  | -0.485 | 0.057 | 0.110 |
| ZNF836 | Q6ZNA1 | Zinc finger protein 836 |  |  | -0.453 | 0.059 | 0.113 |
| PHEX | P78562 | Phosphate-regulating neutral endopeptidase |  |  | -0.574 | 0.062 | 0.117 |
| XIRP2 | A4UGR9 | Xin actin-binding repeat-containing protein 2 |  |  | -0.662 | 0.062 | 0.116 |
| NIPBL | Q6KC79 | Nipped-B-like protein |  |  | 0.481 | 0.063 | 0.117 |
| ZGRF1 | Q86YA3 | Protein ZGRF1 |  |  | -0.361 | 0.069 | 0.128 |
| GCC2 | Q8IWJ2 | GRIP and coiled-coil domain-containing protein 2 |  |  | 0.566 | 0.070 | 0.129 |
| KRT9 | P35527 | Keratin, type I cytoskeletal 9 |  |  | -0.513 | 0.072 | 0.130 |
| ZFHX4 | Q86UP3 | Zinc finger homeobox protein 4 |  |  | -0.418 | 0.073 | 0.132 |
| MYOZ1 | Q9NP98 | Myozenin-1 |  |  | -0.215 | 0.075 | 0.135 |
| CRAT | P43155 | Carnitine O=-acetyltransferase |  |  | 0.382 | 0.075 | 0.134 |
| ANKRD36C | Q5JPF3 | Ankyrin repeat domain-containing protein 36C |  |  | 0.460 | 0.077 | 0.136 |
| NEXN | Q0ZGT2 | Nexilin |  |  | -0.255 | 0.087 | 0.156 |
| CUL4B | Q13620 | Cullin-4B |  |  | -0.386 | 0.091 | 0.160 |
| TMF1 | P82094 | TATA element modulatory factor |  |  | -0.593 | 0.091 | 0.160 |
| CCDC88A | Q3V6T2 | Girdin |  |  | -0.402 | 0.092 | 0.160 |
| CAMK2A | Q9UQM7 | Calcium/calmodulin-dependent protein kinase type II subunit alpha |  |  | 0.328 | 0.094 | 0.161 |
| POTEI | P0CG38 | POTE ankyrin domain family member I |  |  | -0.458 | 0.097 | 0.165 |
| CRYBG3 | Q68DQ2 | Very large A-kinase anchor protein |  |  | 0.436 | 0.104 | 0.177 |
| PLD5 | Q8N7P1 | Inactive phospholipase D5 |  |  | 0.333 | 0.109 | 0.184 |
| HIST1H2BM | Q99879 | Histone H2B type 1-M |  |  | -0.521 | 0.112 | 0.187 |
| CMYA5 | Q8N3K9 | Cardiomyopathy-associated protein 5 |  |  | -0.342 | 0.113 | 0.188 |
| DNAH17 | Q9UFH2 | Dynein heavy chain 17, axonemal |  |  | -0.576 | 0.121 | 0.201 |
| KRT1 | P04264 | Keratin, type II cytoskeletal 1 |  |  | -0.378 | 0.129 | 0.213 |
| ENO1 | P06733 | Alpha-enolase |  |  | -0.320 | 0.131 | 0.216 |
| DNAH2 | Q9P225 | Dynein heavy chain 2, axonemal |  |  | -0.328 | 0.139 | 0.227 |
| KIAA1217 | Q5T5P2 | Sickle tail protein homolog |  |  | -0.433 | 0.139 | 0.225 |
| LAMA2 | P24043 | Laminin subunit alpha-2 |  |  | 0.602 | 0.141 | 0.227 |
| ZZEF1 | O43149 | Zinc finger ZZ-type and EF-hand domain-containing protein 1 |  |  | -0.442 | 0.144 | 0.229 |
| RTN4 | Q9NQC3 | Reticulon-4 |  |  | -0.521 | 0.145 | 0.230 |
| ANKRD11 | Q6UB99 | Ankyrin repeat domain-containing protein 11 |  |  | 0.387 | 0.147 | 0.230 |
| ALMS1 | Q8TCU4 | Alstrom syndrome protein 1 |  |  | -0.500 | 0.153 | 0.238 |
| CKM | P06732 | Creatine kinase M-type |  |  | -0.243 | 0.160 | 0.247 |
| FRMPD3 | Q5JV73 | FERM and PDZ domain-containing protein 3 |  |  | -0.473 | 0.171 | 0.261 |
| UBR4 | Q5T4S7 | E3 ubiquitin-protein ligase UBR4 |  |  | -0.312 | 0.187 | 0.285 |
| DCTN6 | O00399 | Dynactin subunit 6 |  |  | -0.203 | 0.189 | 0.287 |
| GOLGB1 | Q14789 | Golgin subfamily B member 1 |  |  | 0.330 | 0.191 | 0.287 |
| DSP | P15924 | Desmoplakin |  |  | -0.289 | 0.193 | 0.289 |
| SRL | Q86TD4 | Sarcalumenin |  |  | -0.241 | 0.206 | 0.305 |
| NWD2 | Q9ULI1 | NACHT and WD repeat domain-containing protein 2 |  |  | 0.200 | 0.216 | 0.318 |
| PRDM2 | Q13029 | PR domain zinc finger protein 2 |  |  | -0.244 | 0.218 | 0.319 |
| MARVELD2 | Q8N4S9 | MARVEL domain-containing protein 2 |  |  | -0.238 | 0.234 | 0.340 |
| DNAH3 | Q8TD57 | Dynein heavy chain 3, axonemal |  |  | 0.254 | 0.239 | 0.346 |
| ALPK2 | Q86TB3 | Alpha-protein kinase 2 |  |  | 0.374 | 0.242 | 0.347 |
| SPEN | Q96T58 | Msx2-interacting protein |  |  | -0.296 | 0.243 | 0.346 |
| KMT2A | Q03164 | Histone-lysine N-methyltransferase 2A |  |  | 0.359 | 0.252 | 0.358 |
| AKAP9 | Q99996 | A-kinase anchor protein 9 |  |  | 0.389 | 0.264 | 0.372 |
| CASQ1 | P31415 | Calsequestrin-1 |  |  | 0.198 | 0.277 | 0.389 |
| HIST1H4A | P62805 | Histone H4 |  |  | -0.260 | 0.277 | 0.387 |
| RYR3 | Q15413 | Ryanodine receptor 3 |  |  | -0.385 | 0.280 | 0.390 |
| FASN | P49327 | Fatty acid synthase |  |  | 0.206 | 0.281 | 0.389 |
| VPS13A | Q96RL7 | Vacuolar protein sorting-associated protein 13A |  |  | 0.252 | 0.296 | 0.409 |
| ALDOA | P04075 | Fructose-bisphosphate aldolase A |  |  | 0.283 | 0.297 | 0.407 |
| MYH7 | P12883 | Myosin-7 |  |  | -0.364 | 0.297 | 0.405 |
| HECTD4 | Q9Y4D8 | Probable E3 ubiquitin-protein ligase HECTD4 |  |  | -0.327 | 0.305 | 0.413 |
| SRRM2 | Q9UQ35 | Serine/arginine repetitive matrix protein 2 |  |  | -0.372 | 0.326 | 0.442 |
| PLEC | Q15149 | Plectin |  |  | -0.277 | 0.328 | 0.442 |
| SOS2 | Q07890 | Son of sevenless homolog 2 |  |  | -0.225 | 0.337 | 0.453 |
| DNAH5 | Q8TE73 | Dynein heavy chain 5, axonemal |  |  | -0.288 | 0.345 | 0.462 |
| SHROM3 | Q8TF72 | Protein Shroom3 |  |  | 0.366 | 0.347 | 0.462 |
| MACF1 | Q9UPN3 | Microtubule-actin cross-linking factor 1, isoforms 1/2/3/5 |  |  | 0.231 | 0.353 | 0.467 |
| KIF21B | O75037 | Kinesin-like protein KIF21B |  |  | -0.310 | 0.377 | 0.499 |
| TTC27 | Q6P3X3 | Tetratricopeptide repeat protein 27 |  |  | -0.269 | 0.389 | 0.513 |
| DMD | P11532 | Dystrophin |  |  | -0.242 | 0.395 | 0.519 |
| MYH13 | Q9UKX3 | Myosin-13 |  |  | 0.237 | 0.396 | 0.517 |
| MYT1L | Q9UL68 | Myelin transcription factor 1-like protein |  |  | 0.266 | 0.405 | 0.527 |
| FAT4 | Q6V0I7 | Protocadherin Fat 4 |  |  | -0.293 | 0.413 | 0.536 |
| DOCK2 | Q92608 | Dedicator of cytokinesis protein 2 |  |  | -0.224 | 0.423 | 0.547 |
| DST | Q03001 | Dystonin |  |  | -0.105 | 0.446 | 0.574 |
| SPTBN1 | Q01082 | Spectrin beta chain, non-erythrocytic 1 |  |  | -0.199 | 0.460 | 0.589 |
| DES | P17661 | Desmin |  |  | 0.194 | 0.468 | 0.598 |
| DENND4C | Q5VZ89 | DENN domain-containing protein 4C |  |  | 0.153 | 0.468 | 0.595 |
| MYO3A | Q8NEV4 | Myosin-IIIa |  |  | -0.185 | 0.475 | 0.600 |
| CA3 | P07451 | Carbonic anhydrase 3 |  |  | -0.140 | 0.475 | 0.597 |
| ABCA5 | Q8WWZ7 | ATP-binding cassette sub-family A member 5 |  |  | -0.194 | 0.480 | 0.600 |
| SNRNP200 | O75643 | U5 small nuclear ribonucleoprotein 200 kDa helicase |  |  | -0.144 | 0.481 | 0.597 |
| ARL6IP5 | O75915 | PRA1 family protein 3 |  |  | -0.157 | 0.482 | 0.596 |
| MUC16 | Q8WXI7 | Mucin-16 |  |  | -0.257 | 0.483 | 0.594 |
| NCOR1 | O75376 | Nuclear receptor corepressor 1 |  |  | 0.301 | 0.491 | 0.601 |
| BIRC6 | Q9NR09 | Baculoviral IAP repeat-containing protein 6 |  |  | -0.226 | 0.501 | 0.610 |
| CFAP54 | Q96N23 | Cilia- and flagella-associated protein 54 |  |  | 0.173 | 0.522 | 0.636 |
| MAST4 | O15021 | Microtubule-associated serine/threonine-protein kinase 4 |  |  | -0.218 | 0.527 | 0.638 |
| CENPE | Q02224 | Centromere-associated protein E |  |  | 0.176 | 0.533 | 0.642 |
| TPM1 | P09493 | Tropomyosin alpha-1 chain |  |  | 0.099 | 0.545 | 0.654 |
| FGD6 | Q6ZV73 | FYVE, RhoGEF and PH domain-containing protein 6 |  |  | 0.209 | 0.554 | 0.662 |
| PIKFYVE | Q9Y2I7 | 1-phosphatidylinositol 3-phosphate 5-kinase |  |  | 0.123 | 0.559 | 0.666 |
| UNC13A | Q9UPW8 | Protein unc-13 homolog A |  |  | 0.223 | 0.563 | 0.667 |
| MYOF | Q9NZM1 | Myoferlin |  |  | 0.164 | 0.570 | 0.674 |
| MICAL3 | Q7RTP6 | [F-actin]-monooxygenase MICAL3 |  |  | -0.109 | 0.576 | 0.677 |
| AHNAK | Q09666 | Neuroblast differentiation-associated protein AHNAK |  |  | 0.102 | 0.586 | 0.686 |
| MCM8 | Q9UJA3 | DNA helicase MCM8 |  |  | 0.113 | 0.595 | 0.695 |
| SACS | Q9NZJ4 | Sacsin |  |  | 0.153 | 0.602 | 0.700 |
| REV3L | O60673 | DNA polymerase zeta catalytic subunit |  |  | -0.153 | 0.628 | 0.729 |
| PARD3 | Q8TEW0 | Partitioning defective 3 homolog |  |  | -0.092 | 0.633 | 0.732 |
| PCLO | Q9Y6V0 | Protein piccolo |  |  | -0.156 | 0.634 | 0.730 |
| CCDC27 | Q2M243 | Coiled-coil domain-containing protein 27 |  |  | -0.131 | 0.643 | 0.737 |
| FYCO1 | Q9BQS8 | FYVE and coiled-coil domain-containing protein 1 |  |  | -0.108 | 0.648 | 0.740 |
| PDZD2 | O15018 | PDZ domain-containing protein 2 |  |  | 0.107 | 0.650 | 0.737 |
| AHNAK2 | Q8IVF2 | Protein AHNAK2 |  |  | 0.118 | 0.650 | 0.734 |
| LMNA | P02545 | Prelamin-A/C |  |  | -0.121 | 0.650 | 0.731 |
| CEP350 | Q5VT06 | Centrosome-associated protein 350 |  |  | 0.112 | 0.657 | 0.735 |
| MYO1 | O43795 | Unconventional myosin-Ib |  |  | -0.096 | 0.659 | 0.734 |
| RB1CC1 | Q8TDY2 | RB1-inducible coiled-coil protein 1 |  |  | -0.130 | 0.671 | 0.745 |
| MYO15A | Q9UKN7 | Unconventional myosin-XV |  |  | 0.107 | 0.677 | 0.749 |
| ACAT1 | P24752 | Acetyl-CoA acetyltransferase, mitochondrial |  |  | 0.093 | 0.679 | 0.747 |
| DNAH11 | Q96DT5 | Dynein heavy chain 11, axonemal |  |  | -0.128 | 0.689 | 0.756 |
| SYPL2 | Q5VXT5 | Synaptophysin-like protein 2 |  |  | 0.094 | 0.690 | 0.753 |
| PDCD11 | Q14690 | Protein RRP5 homolog |  |  | 0.094 | 0.700 | 0.760 |
| ICE2 | Q659A1 | Little elongation complex subunit 2 |  |  | -0.102 | 0.703 | 0.761 |
| MDN1 | Q9NU22 | Midasin |  |  | 0.131 | 0.709 | 0.763 |
| TOPAZ1 | Q8N9V7 | Protein TOPAZ1 |  |  | -0.107 | 0.713 | 0.764 |
| ABCA13 | Q86UQ4 | ATP-binding cassette sub-family A member 13 |  |  | 0.073 | 0.743 | 0.796 |
| LDB3 | O75112 | LIM domain-binding protein 3 |  |  | -0.060 | 0.765 | 0.817 |
| LAMA3 | Q16787 | Laminin subunit alpha-3 |  |  | -0.094 | 0.766 | 0.814 |
| CADPS | Q9ULU8 | Calcium-dependent secretion activator 1 |  |  | 0.067 | 0.770 | 0.815 |
| KRT10 | P13645 | Keratin, type I cytoskeletal 10 |  |  | 0.086 | 0.805 | 0.852 |
| NAV2 | Q8IVL1 | Neuron navigator 2 |  |  | -0.050 | 0.809 | 0.853 |
| UTRN | P46939 | Utrophin |  |  | -0.064 | 0.829 | 0.872 |
| KIF20B | Q96Q89 | Kinesin-like protein KIF20B |  |  | 0.066 | 0.848 | 0.890 |
| TRIO | O75962 | Triple functional domain protein |  |  | 0.036 | 0.872 | 0.913 |
| AACS | Q86V21 | Acetoacetyl-CoA synthetase |  |  | 0.072 | 0.872 | 0.909 |
| CFAP57 | Q96MR6 | Cilia- and flagella-associated protein 57 |  |  | 0.031 | 0.873 | 0.906 |
| FAM120C | Q9NX05 | Constitutive coactivator of PPAR-gamma-like protein 2 |  |  | -0.041 | 0.877 | 0.907 |
| DOCK9 | Q9BZ29 | Dedicator of cytokinesis protein 9 |  |  | 0.029 | 0.896 | 0.924 |
| KMT2D | O14686 | Histone-lysine N-methyltransferase 2D |  |  | 0.038 | 0.901 | 0.926 |
| DNAH8 | Q96JB1 | Dynein heavy chain 8, axonemal |  |  | 0.024 | 0.910 | 0.931 |
| MKI67 | P46013 | Proliferation marker protein Ki-67 |  |  | -0.029 | 0.924 | 0.942 |
| VPS13B | Q7Z7G8 | Vacuolar protein sorting-associated protein 13B |  |  | 0.015 | 0.944 | 0.960 |
| FANCM | Q8IYD8 | Fanconi anemia group M protein |  |  | -0.012 | 0.960 | 0.974 |
| DNAH10 | Q8IVF4 | Dynein heavy chain 10, axonemal |  |  | 0.008 | 0.964 | 0.975 |
| CKMT2 | P17540 | Creatine kinase S-type, mitochondrial |  |  | -0.010 | 0.983 | 0.990 |
| KRT6B | P04259 | Keratin, type II cytoskeletal 6B |  |  | -0.004 | 0.991 | 0.995 |
| KRT14 | P02533 | Keratin, type I cytoskeletal 14 |  |  | -0.004 | 0.991 | 0.991 |
| TRPM2 | O94759 | Transient receptor potential cation channel subfamily M member 2 |  |  | -0.495 | 0.052 | 0.102 |
| TTC3 | P53804 | E3 ubiquitin-protein ligase TTC3 |  |  | 0.428 | 0.054 | 0.106 |
| KIAA1107 | Q9UPP5 | Uncharacterized protein KIAA1107 |  |  | 0.509 | 0.055 | 0.107 |
| MB | P02144 | Myoglobin |  |  | -0.485 | 0.057 | 0.110 |
| ZNF836 | Q6ZNA1 | Zinc finger protein 836 |  |  | -0.453 | 0.059 | 0.113 |
| PHEX | P78562 | Phosphate-regulating neutral endopeptidase |  |  | -0.574 | 0.062 | 0.117 |
| XIRP2 | A4UGR9 | Xin actin-binding repeat-containing protein 2 |  |  | -0.662 | 0.062 | 0.116 |
| NIPBL | Q6KC79 | Nipped-B-like protein |  |  | 0.481 | 0.063 | 0.117 |
| ZGRF1 | Q86YA3 | Protein ZGRF1 |  |  | -0.361 | 0.069 | 0.128 |
| GCC2 | Q8IWJ2 | GRIP and coiled-coil domain-containing protein 2 |  |  | 0.566 | 0.070 | 0.129 |
| KRT9 | P35527 | Keratin, type I cytoskeletal 9 |  |  | -0.513 | 0.072 | 0.130 |
| ZFHX4 | Q86UP3 | Zinc finger homeobox protein 4 |  |  | -0.418 | 0.073 | 0.132 |
| MYOZ1 | Q9NP98 | Myozenin-1 |  |  | -0.215 | 0.075 | 0.135 |
| CRAT | P43155 | Carnitine O=-acetyltransferase |  |  | 0.382 | 0.075 | 0.134 |
| ANKRD36C | Q5JPF3 | Ankyrin repeat domain-containing protein 36C |  |  | 0.460 | 0.077 | 0.136 |
| NEXN | Q0ZGT2 | Nexilin |  |  | -0.255 | 0.087 | 0.156 |
| CUL4B | Q13620 | Cullin-4B |  |  | -0.386 | 0.091 | 0.160 |
| TMF1 | P82094 | TATA element modulatory factor |  |  | -0.593 | 0.091 | 0.160 |
| CCDC88A | Q3V6T2 | Girdin |  |  | -0.402 | 0.092 | 0.160 |
| CAMK2A | Q9UQM7 | Calcium/calmodulin-dependent protein kinase type II subunit alpha |  |  | 0.328 | 0.094 | 0.161 |
| POTEI | P0CG38 | POTE ankyrin domain family member I |  |  | -0.458 | 0.097 | 0.165 |
| CRYBG3 | Q68DQ2 | Very large A-kinase anchor protein |  |  | 0.436 | 0.104 | 0.177 |
| PLD5 | Q8N7P1 | Inactive phospholipase D5 |  |  | 0.333 | 0.109 | 0.184 |
| HIST1H2BM | Q99879 | Histone H2B type 1-M |  |  | -0.521 | 0.112 | 0.187 |
| CMYA5 | Q8N3K9 | Cardiomyopathy-associated protein 5 |  |  | -0.342 | 0.113 | 0.188 |
| DNAH17 | Q9UFH2 | Dynein heavy chain 17, axonemal |  |  | -0.576 | 0.121 | 0.201 |
| KRT1 | P04264 | Keratin, type II cytoskeletal 1 |  |  | -0.378 | 0.129 | 0.213 |
| ENO1 | P06733 | Alpha-enolase |  |  | -0.320 | 0.131 | 0.216 |
| DNAH2 | Q9P225 | Dynein heavy chain 2, axonemal |  |  | -0.328 | 0.139 | 0.227 |
| KIAA1217 | Q5T5P2 | Sickle tail protein homolog |  |  | -0.433 | 0.139 | 0.225 |
| LAMA2 | P24043 | Laminin subunit alpha-2 |  |  | 0.602 | 0.141 | 0.227 |
| ZZEF1 | O43149 | Zinc finger ZZ-type and EF-hand domain-containing protein 1 |  |  | -0.442 | 0.144 | 0.229 |
| RTN4 | Q9NQC3 | Reticulon-4 |  |  | -0.521 | 0.145 | 0.230 |
| ANKRD11 | Q6UB99 | Ankyrin repeat domain-containing protein 11 |  |  | 0.387 | 0.147 | 0.230 |
| ALMS1 | Q8TCU4 | Alstrom syndrome protein 1 |  |  | -0.500 | 0.153 | 0.238 |
| CKM | P06732 | Creatine kinase M-type |  |  | -0.243 | 0.160 | 0.247 |
| FRMPD3 | Q5JV73 | FERM and PDZ domain-containing protein 3 |  |  | -0.473 | 0.171 | 0.261 |
| UBR4 | Q5T4S7 | E3 ubiquitin-protein ligase UBR4 |  |  | -0.312 | 0.187 | 0.285 |
| DCTN6 | O00399 | Dynactin subunit 6 |  |  | -0.203 | 0.189 | 0.287 |
| GOLGB1 | Q14789 | Golgin subfamily B member 1 |  |  | 0.330 | 0.191 | 0.287 |
| DSP | P15924 | Desmoplakin |  |  | -0.289 | 0.193 | 0.289 |
| SRL | Q86TD4 | Sarcalumenin |  |  | -0.241 | 0.206 | 0.305 |
| NWD2 | Q9ULI1 | NACHT and WD repeat domain-containing protein 2 |  |  | 0.200 | 0.216 | 0.318 |
| PRDM2 | Q13029 | PR domain zinc finger protein 2 |  |  | -0.244 | 0.218 | 0.319 |
| MARVELD2 | Q8N4S9 | MARVEL domain-containing protein 2 |  |  | -0.238 | 0.234 | 0.340 |
| DNAH3 | Q8TD57 | Dynein heavy chain 3, axonemal |  |  | 0.254 | 0.239 | 0.346 |
| ALPK2 | Q86TB3 | Alpha-protein kinase 2 |  |  | 0.374 | 0.242 | 0.347 |
| SPEN | Q96T58 | Msx2-interacting protein |  |  | -0.296 | 0.243 | 0.346 |
| KMT2A | Q03164 | Histone-lysine N-methyltransferase 2A |  |  | 0.359 | 0.252 | 0.358 |
| DNAH8 | Q96JB1 | Dynein heavy chain 8, axonemal |  |  | 0.389 | 0.264 | 0.372 |
| MKI67 | P46013 | Proliferation marker protein Ki-67 |  |  | 0.198 | 0.277 | 0.389 |
| VPS13B | Q7Z7G8 | Vacuolar protein sorting-associated protein 13B |  |  | -0.260 | 0.277 | 0.387 |
| FANCM | Q8IYD8 | Fanconi anemia group M protein |  |  | -0.385 | 0.280 | 0.390 |
| DNAH10 | Q8IVF4 | Dynein heavy chain 10, axonemal |  |  | 0.206 | 0.281 | 0.389 |
| CKMT2 | P17540 | Creatine kinase S-type, mitochondrial |  |  | 0.252 | 0.296 | 0.409 |
| KRT6B | P04259 | Keratin, type II cytoskeletal 6B |  |  | 0.283 | 0.297 | 0.407 |
| KRT14 | P02533 | Keratin, type I cytoskeletal 14 |  |  | -0.364 | 0.297 | 0.405 |

Supplementary table 2

**Proteins assigned to functional clusters detected in manually dissected fibers originating from CFZS patients and healthy controls.** Metascape determined both enrichment and p values for protein clusters and associated proteins. Visual representation of pathway clusters found in figure 5.

| *Cluster name* | *Protein number* | *Proteins in cluster* | *Up in CON or CFZS* | *Best enrich-ment in cluster* | *Best Log p in cluster* |
| --- | --- | --- | --- | --- | --- |
| *Cardiac muscle contraction* | 11 | Sarcoplasmic/endoplasmic reticulum calcium ATPase 2 | CON | 139.03 | -16.57 |
|  |  | Cytochrome c oxidase subunit 4 isoform 1, mitochondrial |  |  |  |
|  |  | Cytochrome c oxidase subunit 5B, mitochondrial |  |  |  |
|  |  | Cytochrome c1, heme protein, mitochondrial |  |  |  |
|  |  | Cytochrome c oxidase subunit 2 |  |  |  |
|  |  | Myosin regulatory light chain 2, ventricular/cardiac muscle isoform |  |  |  |
|  |  | Tropomyosin beta chain |  |  |  |
|  |  | Tropomyosin alpha-3 chain |  |  |  |
|  |  | Cytochrome b-c1 complex subunit 7 |  |  |  |
|  |  | Cytochrome b-c1 complex subunit 1, mitochondrial |  |  |  |
|  |  | Cytochrome b-c1 complex subunit 2, mitochondrial |  |  |  |
| *F1F0-ATP synthase, mitochondrial* | 6 | ATP synthase subunit alpha, mitochondrial | CON | 91.24 | -5.34 |
|  |  | ATP synthase subunit beta, mitochondrial |  |  |  |
|  |  | ATP synthase subunit gamma, mitochondrial |  |  |  |
|  |  | Neurofibromin |  |  |  |
|  |  | Centrosomal protein of 135 kDa |  |  |  |
|  |  | Nuclear autoantigen Sp-100 |  |  |  |
| mitochondrial electron transport, cytochrome c to oxygen | 4 | Cytochrome c oxidase subunit 4 isoform 1, mitochondrial | CON | 81.10 | -6.78 |
|  |  | Cytochrome c oxidase subunit 5B, mitochondrial |  |  |  |
|  |  | Cytochrome c oxidase subunit 2 |  |  |  |
|  |  | Cytochrome c oxidase subunit NDUFA4 |  |  |  |
| cellular respiration | 22 | ADP/ATP translocase 1 | CON | 70.86 | -24.73 |
|  |  | ATP synthase subunit alpha, mitochondrial |  |  |  |
|  |  | NADH-ubiquinone oxidoreductase 75 kDa subunit, mitochondrial |  |  |  |
|  |  | NADH dehydrogenase [ubiquinone] iron-sulfur protein 3, mitochondrial |  |  |  |
|  |  | Succinate dehydrogenase [ubiquinone] flavoprotein subunit, mitochondrial |  |  |  |
|  |  | Inositol 1,4,5-trisphosphate receptor type 1 |  |  |  |
|  |  | Cytochrome c oxidase subunit 2 |  |  |  |
|  |  | Cytochrome c1, heme protein, mitochondrial |  |  |  |
|  |  | Cytochrome c oxidase subunit NDUFA4 |  |  |  |
|  |  | Cytochrome c oxidase subunit 4 isoform 1, mitochondrial |  |  |  |
|  |  | Cytochrome c oxidase subunit 5B, mitochondrial |  |  |  |
|  |  | ATP synthase subunit gamma, mitochondrial |  |  |  |
|  |  | Cytochrome b-c1 complex subunit 1, mitochondrial |  |  |  |
|  |  | Cytochrome b-c1 complex subunit 2, mitochondrial |  |  |  |
|  |  | Voltage-dependent anion-selective channel protein 1 |  |  |  |
|  |  | ATP synthase subunit beta, mitochondrial |  |  |  |
|  |  | Sarcoplasmic/endoplasmic reticulum calcium ATPase 2 |  |  |  |
|  |  | Cytochrome b-c1 complex subunit 7 |  |  |  |
|  |  | NADH dehydrogenase [ubiquinone] 1 beta subcomplex subunit 4 |  |  |  |
|  |  | Calcium-binding mitochondrial carrier protein Aralar1 |  |  |  |
|  |  | NAD(P) transhydrogenase, mitochondrial |  |  |  |
|  |  | Protein NipSnap homolog 2 |  |  |  |
| *Striated Muscle Contraction* | 10 | Actin, alpha skeletal muscle | CON | 67.58 | -7.95 |
|  |  | Sarcoplasmic/endoplasmic reticulum calcium ATPase 2 |  |  |  |
|  |  | Neurofibromin |  |  |  |
|  |  | Troponin I, slow skeletal muscle |  |  |  |
|  |  | Tropomyosin beta chain |  |  |  |
|  |  | PDZ and LIM domain protein 5 |  |  |  |
|  |  | Myosin regulatory light chain 2, ventricular/cardiac muscle isoform |  |  |  |
|  |  | Tropomyosin alpha-3 chain |  |  |  |
|  |  | Unconventional myosin-XVIIIb |  |  |  |
|  |  | Inositol 1,4,5-trisphosphate receptor type 1 |  |  |  |
| *Mitochondrial protein import* | 12 | ADP/ATP translocase 1 | CON | 45.62 | -8.40 |
|  |  | Neurofibromin |  |  |  |
|  |  | Sarcoplasmic/endoplasmic reticulum calcium ATPase 2 |  |  |  |
|  |  | Phosphate carrier protein, mitochondrial |  |  |  |
|  |  | Inositol 1,4,5-trisphosphate receptor type 1 |  |  |  |
|  |  | ATP synthase subunit alpha, mitochondrial |  |  |  |
|  |  | Voltage-dependent anion-selective channel protein 1 |  |  |  |
|  |  | Calcium-binding mitochondrial carrier protein Aralar1 |  |  |  |
|  |  | ATP synthase subunit beta, mitochondrial |  |  |  |
|  |  | G2/mitotic-specific cyclin-B3 |  |  |  |
|  |  | Cytochrome c1, heme protein, mitochondrial |  |  |  |
|  |  | NADH-cytochrome b5 reductase 1 |  |  |  |
| *proton transmembrane transport* | 14 | ADP/ATP translocase 1 | CON | 38.79 | -14.30 |
|  |  | Sarcoplasmic/endoplasmic reticulum calcium ATPase 2 |  |  |  |
|  |  | ATP synthase subunit alpha, mitochondrial |  |  |  |
|  |  | ATP synthase subunit beta, mitochondrial |  |  |  |
|  |  | ATP synthase subunit gamma, mitochondrial |  |  |  |
|  |  | Cytochrome c oxidase subunit 4 isoform 1, mitochondrial |  |  |  |
|  |  | Cytochrome c oxidase subunit 5B, mitochondrial |  |  |  |
|  |  | Cytochrome c1, heme protein, mitochondrial |  |  |  |
|  |  | Inositol 1,4,5-trisphosphate receptor type 1 |  |  |  |
|  |  | Cytochrome c oxidase subunit 2 |  |  |  |
|  |  | Phosphate carrier protein, mitochondrial |  |  |  |
|  |  | Cytochrome b-c1 complex subunit 1, mitochondrial |  |  |  |
|  |  | NAD(P) transhydrogenase, mitochondrial |  |  |  |
|  |  | Magnesium transporter NIPA4 |  |  |  |
| *Pyruvate metabolism and Citric Acid (TCA) cycle*  *organelle assembly* | 3 | Succinate dehydrogenase [ubiquinone] flavoprotein subunit, mitochondrial | CON | 26.54 | -3.70 |
|  |  | Voltage-dependent anion-selective channel protein 1 |  |  |  |
|  |  | NAD(P) transhydrogenase, mitochondrial |  |  |  |
| *organelle assembly* | 12 | Nuclear mitotic apparatus protein 1 | CON | 17.80 | -3.67 |
|  |  | Oxygen-regulated protein 1 |  |  |  |
|  |  | Centrosomal protein of 135 kDa |  |  |  |
|  |  | Dynein assembly factor 1, axonemal |  |  |  |
|  |  | Abnormal spindle-like microcephaly-associated protein |  |  |  |
|  |  | Semaphorin-4D |  |  |  |
|  |  | Probable helicase senataxin |  |  |  |
|  |  | ADP/ATP translocase 1 |  |  |  |
|  |  | Voltage-dependent anion-selective channel protein 1 |  |  |  |
|  |  | Actin, alpha skeletal muscle |  |  |  |
|  |  | Sarcoplasmic/endoplasmic reticulum calcium ATPase 2 |  |  |  |
|  |  | Myosin regulatory light chain 2, ventricular/cardiac muscle isoform |  |  |  |
| *negative regulation of cell growth* | 3 | Myosin regulatory light chain 2, ventricular/cardiac muscle isoform | CON | 7.76 | -2.16 |
|  |  | NADH dehydrogenase [ubiquinone] iron-sulfur protein 3, mitochondrial |  |  |  |
|  |  | Semaphorin-4D |  |  |  |
| *regulation of apoptotic signaling pathway* | 5 | ADP/ATP translocase 1 | CON | 7.19 | -2.13 |
|  |  | NADH dehydrogenase [ubiquinone] iron-sulfur protein 3, mitochondrial |  |  |  |
|  |  | Neurofibromin |  |  |  |
|  |  | Nuclear autoantigen Sp-100 |  |  |  |
|  |  | E3 ubiquitin-protein ligase HECTD1 |  |  |  |
| *actin-myosin filament sliding* | 3 | \| Myosin-2 \| \| --- \| \| Myosin light chain 1/3, skeletal muscle isoform \| \| Myosin regulatory light chain 2, skeletal muscle isoform \| | CFZS | 204.77 | -6.43 |
| *Striated Muscle Contraction* | 16 | \| Alpha-actinin-2 \| \| --- \| \| Myosin-binding protein C, slow-type \| \| Myosin light chain 1/3, skeletal muscle isoform \| \| Nebulin \| \| Troponin I, fast skeletal muscle \| \| Troponin T, fast skeletal muscle \| \| Titin \| \| Filamin-C \| \| Myomesin-2 \| \| Kelch-like protein 41 \| \| Sarcoplasmic/endoplasmic reticulum calcium ATPase 1 \| \| Myosin regulatory light chain 2, skeletal muscle isoform \| \| Myc box-dependent-interacting protein 1 \| \| Alpha-crystallin B chain \| \| Supervillin \| \| Nesprin-1 \| | CFZS | 172.54 | -14.01 |
| *protein refolding* | 3 | Alpha-crystallin B chain | CFZS | 106.48 | -5.53 |
|  |  | Stress-70 protein, mitochondrial |  |  |  |
|  |  | Heat shock protein beta-1 |  |  |  |
| *muscle system process* | 11 | Alpha-crystallin B chain | CFZS | 38.58 | -13.95 |
|  |  | Myosin-1 |  |  |  |
|  |  | Myosin-2 |  |  |  |
|  |  | Myosin light chain 1/3, skeletal muscle isoform |  |  |  |
|  |  | Troponin I, fast skeletal muscle |  |  |  |
|  |  | Troponin T, fast skeletal muscle |  |  |  |
|  |  | Titin |  |  |  |
|  |  | Myomesin-2 |  |  |  |
|  |  | Kelch-like protein 41 |  |  |  |
|  |  | Myosin regulatory light chain 2, skeletal muscle isoform |  |  |  |
|  |  | Sarcoplasmic/endoplasmic reticulum calcium ATPase 1 |  |  |  |
| *regulation of muscle contraction* | 4 | Myc box-dependent-interacting protein 1 | CFZS | 27.44 | -4.36 |
|  |  | Sarcoplasmic/endoplasmic reticulum calcium ATPase 1 |  |  |  |
|  |  | Troponin I, fast skeletal muscle |  |  |  |
|  |  | Troponin T, fast skeletal muscle |  |  |  |
| *regulation of actin filament length* | 6 | Alpha-actinin-2 | CFZS | 21.91 | -4.48 |
|  |  | Myc box-dependent-interacting protein 1 |  |  |  |
|  |  | Sarcoplasmic/endoplasmic reticulum calcium ATPase 1 |  |  |  |
|  |  | Nebulin |  |  |  |
|  |  | Supervillin |  |  |  |
|  |  | Alpha-crystallin B chain |  |  |  |
| *muscle organ development* | 7 | Supervillin | CFZS | 20.17 | -5.98 |
|  |  | Alpha-crystallin B chain |  |  |  |
|  |  | Alpha-actinin-2 |  |  |  |
|  |  | Kelch-like protein 41 |  |  |  |
|  |  | Nebulin |  |  |  |
|  |  | Myosin regulatory light chain 2, skeletal muscle isoform |  |  |  |
|  |  | Titin |  |  |  |
| Salmonella infection | 3 | Filamin-C | CFZS | 10.69 | -2.56 |
|  |  | Glyceraldehyde-3-phosphate dehydrogenase |  |  |  |
|  |  | Myosin regulatory light chain 2, skeletal muscle isoform |  |  |  |
| *negative regulation of endopeptidase activity* | 6 | Myc box-dependent-interacting protein 1 | CFZS | 10.56 | -2.55 |
|  |  | Alpha-crystallin B chain |  |  |  |
|  |  | Glyceraldehyde-3-phosphate dehydrogenase |  |  |  |
|  |  | Ubiquitin carboxyl-terminal hydrolase 4 |  |  |  |
|  |  | Heat shock protein beta-1 |  |  |  |
|  |  | Treacle protein |  |  |  |
